# Structural insight into negative DNA supercoiling by DNA gyrase, a bacterial type 2A DNA topoisomerase

**DOI:** 10.1093/nar/gkt560

**Published:** 2013-06-26

**Authors:** Julie Papillon, Jean-François Ménétret, Claire Batisse, Reynald Hélye, Patrick Schultz, Noëlle Potier, Valérie Lamour

**Affiliations:** ^1^IGBMC, Integrated Structural Biology Department, UMR7104 CNRS, U964 Inserm, Université de Strasbourg, 67400 Illkirch, France, ^2^Institut de Chimie de Strasbourg, Université de Strasbourg, UMR7177 CNRS, 67000 Strasbourg, France and ^3^Hôpitaux Universitaires de Strasbourg, 67000 Strasbourg, France

## Abstract

Type 2A DNA topoisomerases (Topo2A) remodel DNA topology during replication, transcription and chromosome segregation. These multisubunit enzymes catalyze the transport of a double-stranded DNA through a transient break formed in another duplex. The bacterial DNA gyrase, a target for broad-spectrum antibiotics, is the sole Topo2A enzyme able to introduce negative supercoils. We reveal here for the first time the architecture of the full-length *Thermus thermophilus* DNA gyrase alone and in a cleavage complex with a 155 bp DNA duplex in the presence of the antibiotic ciprofloxacin, using cryo-electron microscopy. The structural organization of the subunits of the full-length DNA gyrase points to a central role of the ATPase domain acting like a ‘crossover trap’ that may help to sequester the DNA positive crossover before strand passage. Our structural data unveil how DNA is asymmetrically wrapped around the gyrase-specific C-terminal β-pinwheel domains and guided to introduce negative supercoils through cooperativity between the ATPase and β-pinwheel domains. The overall conformation of the drug-induced DNA binding–cleavage complex also suggests that ciprofloxacin traps a DNA pre-transport conformation.

## INTRODUCTION

The essential function of DNA topoisomerases regulates the underwinding or overwinding of DNA that occurs during replication, chromosome segregation or transcription of the intertwined DNA double helix (1,2). To help overcome these topological problems, type 2A DNA topoisomerases (Topo2A) bind and cut the phosphate backbone of double-stranded DNA (dsDNA). This transient break allows the DNA to be relaxed or untangled with resealing at the end of the process (2). These enzymes essential cellular role has stimulated research in the field of antibiotic and anti-cancer drug design for many decades (3,4).

**Figure 1.**
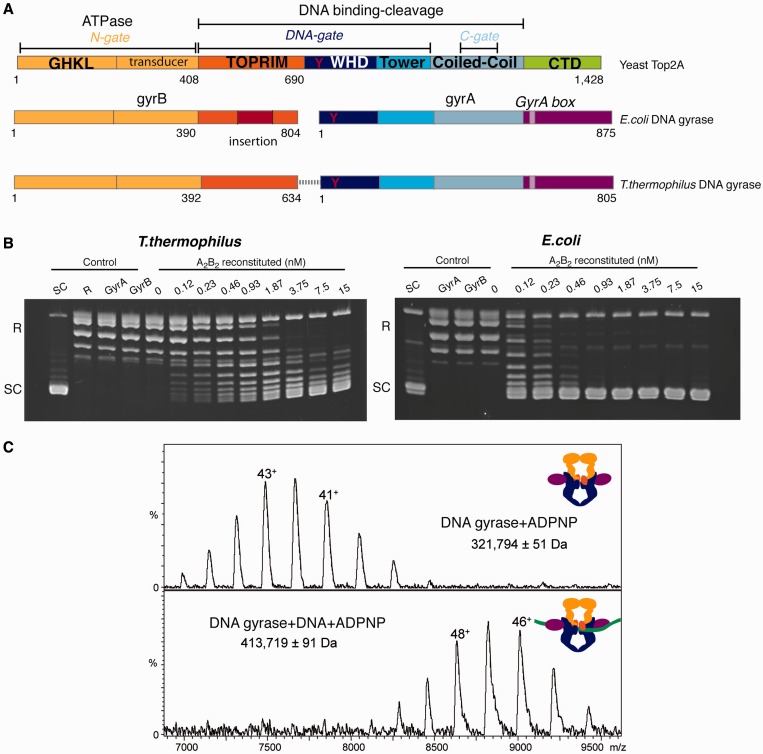
Domain organization and characterization of DNA gyrase complexes. (**A**) Domain organization of the eukaryotic Topo2A and prokaryotic DNA gyrase. Functional regions are colored and labeled. The CTD is divergent between prokaryotes and eukaryotes (*green and purple*). The DNA gyrase CTD contains a conserved GyrA box motif (*lilas box*)*.* The catalytic tyrosine in the WHD domain is shown as a red Y letter and is conserved through evolution. The linker connecting the DNA gyrase A and B subunits used for cryo-EM studies is represented as a gray dash line. (**B**) *E. coli* and *T. thermophilus* negative supercoiling enzyme assays. Increasing concentration of reconstituted *E. coli* or *T. thermophilus* DNA gyrase have been incubated at 37 or 65°C, respectively, with relaxed pUC19 plasmid in presence of 1 mM ATP and run on an agarose gel colored with ethidium bromide. DNA topoisomers are labeled on the left of each gel. SC: supercoiled DNA, R: relaxed DNA. The *T. thermophilus* DNA gyrase displays an optimal supercoiling activity at 65°C and supercoils DNA to a lower extent than the mesophile *E. coli* enzyme in the same concentration range. (**C**) Native mass spectrometry on the holoenzyme and DNA-bound complex showing the presence of the dimeric A_2_B_2_ form of the fusion DNA gyrase in presence of ADPNP and formation of the complex with DNA (green) as shown by the total shift of the mass spectra. The measured masses (in Da) are indicated under the name of each complex.

Topo2A enzymes are largely conserved at the sequence and structural level, allowing the extrapolation of biochemical and structural properties between the bacterial and eukaryotic enzymes. The bacterial Topo2A paralogues DNA gyrase and Topoisomerase IV (TopoIV) are heterotetrameric enzymes composed of two subunits, respectively GyrA/GyrB and ParC/ParE. The eukaryotic Topo2A are homodimers in which the composing subunits correspond to a fused version of the B/E and A/C homologs into a single polypeptide (5). These modular enzymes possess a core set of conserved ATPase (N-gate), DNA binding–cleavage domains (DNA- and C-gate) and an idiosyncratic C-terminal domain (CTD; Figure 1A). The sequence of events leading to DNA relaxation and decatenation first involve the binding in the DNA-gate of a DNA G-segment that is kinked on both sides of the cleavage site (6). The G-segment is cleaved by the reversible nucleophilic attack of conserved tyrosines subsequent to another DNA capture, namely the T-segment, through the N-gate (7). Transport of the T-segment through the G-segment double strand break is coupled to ATP hydrolysis through coordinated opening and closing of the dimer interface in the so-called ‘three-gate mechanism’ that leads to the exit of the T-segment through the C-gate after G-segment religation (8,9).

Previous electron microscopy studies have corroborated the hypothesis that these enzymes can bind DNA crossover on either supercoiled DNA or relaxed plasmid (10). Bacterial and human Topo2A enzymes act preferentially on positive supercoiled DNA (11–13). The quaternary organization of the DNA binding–cleavage domains alone and with a 20–34 bp duplex DNA has been revealed by structural studies and is conserved in all organisms (6,7,14–19). Binding of the G-segment induces a swiveling of the Winged Helix Domain (WHD) and TOpoisomerase-PRIMase (TOPRIM) domains closing on the DNA double helix. This structural rearrangement of the WHD and TOPRIM domains allows the binding of another DNA duplex or T-segment that forms an electrostatically stable positive crossover with the G-segment (20).

As shown in the recent crystal structure of the yeast enzyme, the enzyme quaternary organization involves both the DNA binding–cleavage and the N-terminal ATPase domains (14). However, to this day, the structural details of the interaction of a T- and G-segment DNA crossover with the enzyme cavities in the context of the full-length enzyme remain unknown. In contrast with other Topo2A enzymes, the bacterial DNA gyrase possess the unique ability to introduce negative supercoils into DNA, a specific activity essential for bacterial genome packaging and regulation (21). In addition to the catalytic domains, the CTD plays an essential role in discriminating the DNA crossover geometry and particularly positive crossover during ATP-independent DNA relaxation and DNA supercoiling (22,23). DNA gyrase CTDs display a circular β-pinwheel fold and contribute to its ATP-dependent negative supercoiling activity (24–26). The DNA gyrase β-pinwheel fold is similar to a β-propeller and is composed of six blades forming a positively charged lateral surface where DNA binds. This particular CTD structure is either truncated in the bacterial paralogue TopoIV or absent altogether in other Topo2A enzymes (5,23). Complete CTD deletion in DNA gyrase abolishes both ATP-independent DNA relaxation and DNA supercoiling (25). The first blade of DNA gyrase β-pinwheel bears the conserved sequence motif ‘GyrA-box’ where mutations of conserved residues abolish supercoiling but not DNA relaxation (27).

During DNA supercoiling, the T-segment DNA is contiguous to the central G-segment and wraps around the β-pinwheels to introduce the necessary torsion to form a positive crossover with the G-segment. As shown in DNA footprinting studies, the central DNA oligonucleotide and the flanking regions account for ∼120–150 bp of DNA wrapping around the enzyme (28–30). Several solution studies have highlighted that the CTD domains can adopt multiple positions relative to the DNA gyrase core catalytic domains during the supercoiling cycle (31,32). However, the lack of structural information on the full architecture of this large modular enzyme and the poorly understood path of large DNA duplexes still leaves essential questions unanswered about the spatial organization of DNA gyrase domains leading to intramolecular DNA wrapping. Moreover, although structural data on individual and drug-bound DNA gyrase domains have contributed to decipher the structural details of several antibiotic binding pockets (15,16,18,33), information about the overall enzyme conformation when targeted by drugs has lagged behind.

To elucidate the complete architecture of DNA gyrase, we have determined the structure of the full-length *Thermus thermophilus* DNA gyrase in absence and presence of a 155 bp long DNA blocked with a quinolone antibiotic using supramolecular mass spectrometry and 3D cryo-electron microscopy (cryo-EM). Our work reveals for the first time the quaternary domain organization of a full-length DNA gyrase and the path of a long DNA that asymmetrically wraps around the β-pinwheel CTD. Our data suggest that the ATPase and CTD domains structurally cooperate to capture the T-segment and trap the DNA crossover. In addition, these structural data show that the antibacterial ciprofloxacin targets an ATP-bound state of DNA gyrase trapping a T-segment pre-transport conformation.

## MATERIALS AND METHODS

### Wild-type and fusion DNA gyrase cloning

The coding regions of *Thermus thermophilus* and *Escherichia coli* DNA gyrase (GyrA, GyrB) subunits were amplified from genomic DNA and cloned into pET30a and pET28a vectors, respectively, containing a N-terminal poly-histidine tag (further details are provided in the Supplementary Methods). To simplify structural studies of a tetrameric A_2_B_2_ complex, *T. thermophilus* GyrB and GyrA were fused with a three-amino acid G-D-L linker and cloned into our in-house modified pET28a vector producing a GyrB–GyrA fusion protein (GyrBA-fus) with a N-terminal decahistidine (His10) tag upstream of a PreScission protease cleavage site (P3C, GE Healthcare Life Science).

### Protein and DNA sample preparation

Protein expression was carried out in *E. coli* BL21 (DE3) cells and the protein purification was performed using three chromatographic steps (Nickel affinity, ion exchange and size exclusion chromatographies). The 155 bp DNA covering a major quinolone mediated cleavage site centered on position 990 of plasmid pBR322 (30,34) was cloned in a high copy vector and purified by gel filtration after restriction enzyme digestion. Detailed procedures and buffers composition as well as the DNA relaxation and supercoiling protocols are fully described in Supplementary Methods.

### Mass spectrometry analysis

Before native electrospray ionisation mass spectrometry (ESI-MS) analysis, the GyrBA-fus buffer was exchanged with 200 mM ammonium acetate (pH 7.5) using mini gel filtration columns (Microbiospin 30, Biorad). ESI-MS measurements were performed on an electrospray time-of-flight mass spectrometer (MicrOTOF, Bruker Daltonic, Germany). The capillary exit voltage was set to optimize the desolvation process while preventing dissociation of the non-covalent species (300–400V for the protein alone and the protein/DNA complexes, 150–250V for the protein/ADPNP interactions). Samples were diluted to 8 µM in the same buffer and continuously infused into the ESI ion source at a flow rate of 3 µl/min through a Harvard syringe pump (Harvard Apparatus model 11). For protein/DNA interaction, the 155 bp DNA was mixed to the protein in a 1:1.2 ratio in presence or absence of 5′-adenylyl-beta,gamma-imidodiphosphate (ADPNP).

Matrix-Assisted Laser Desorption Ionisation (MALDI) mass spectrometry procedures of full-length cross-linked DNA gyrase and cross-linked DNA gyrase limited proteolysis are available in the Supplementary Methods.

### Image processing and 3D Reconstruction

Sample and grid preparation for cryo-EM experiments is detailed in the Supplementary Methods. Images of both holoenzyme and DNA-bound enzyme embedded in vitreous buffer were recorded at 59 000× magnification on a Tecnai G2 Polara F30 cryo-EM operated at 100 keV with an Eagle 4k × 4k CCD camera (FEI). Images were recorded in low dose conditions with a specimen dose of 20 electrons/Å^2^ per CCD frame and a pixel size of 1.92 Å/pixel. Data collection was performed automatically using the EPU software (FEI). Images were acquired in a defocus range of −1 to −3 µm.

Particles were selected from individual CCD frames in a semiautomated mode using e2boxer in the EMAN2 package (35,36) at a box size of 192 pixels. The contrast transfer function parameters for each micrograph were determined using EMAN2 (http://blake.bcm.edu/emanwiki/EMAN2/ Programs). Automatically selected particle images were corrected by phase flipping. A total of 62 322 and 65 681 particles for holo and DNA-bound enzymes, respectively, were then processed using EMAN2 as described below.

#### Holoenzyme map 3D reconstruction

Before cryo-EM data collection, several 3D maps were generated by random conical tilt from negatively stained particles and aligned in 3D, reflecting the sample heterogeneity. The 3D models corresponding to different orientations of the particle were averaged to populate the missing cone of information present in each individual 3D model. The most populated conformation corresponded to an elongated particle about 17-nm high and 9-nm large. A low-resolution map of this conformation was then determined from a data set of cryo-negatively stain particles.

The cryo-EM data set without any staining was then analyzed by an iterative protocol consisting of a reference-based classification of particles followed by class averaging and 3D model reconstruction (36,37). The cryo-EM sample displays the same heterogeneity as the negative stained samples suggesting multiple conformations. To separate the different states and resolve the ADPNP-bound form, a multirefine strategy was used to process the cryo-EM data with multiple initial models (3 ab-initio models and the cryo-negative map filtered to 50 Å). From this analysis, the most populated subset with 20 500 particles from 600 CCD frames generated a map that corresponded in size and shape to the elongated particles observed in the negative staining experiments. The reconstruction of this subset was further refined with the cryo-negatively stain map as starting model until convergence was achieved using a final angular step of 3° to obtain the holoenzyme map (ADPNP-bound). The cryo-negative staining map obeyed a 2-fold symmetry. The latter was then applied during the last refinement step. The Fourier Shell Correlation function was calculated by comparing two distinct reconstructions obtained by splitting the data in two sets. The resolution of the final reconstruction was assessed according to the Fourier Shell Correlation criterion (38) at 0.5 (FSC_0.5_) at 16.9 Å (12.5 Å using FSC_0.143_). The final reconstruction was filtered to 16.9 Å. Model fitting using crystal structures of DNA gyrase individual domains is described in Supplementary Methods.

#### DNA-bound map 3D reconstruction

The holoenzyme map was used as an initial model on a subset of ∼24 000 particles of DNA gyrase with DNA to reconstruct a first DNA-bound map that showed additional densities. The holoenzyme and initial DNA-bound map were used as starting models to sort the holoenzyme with no DNA from the DNA-bound enzymes on a larger data set of 65 681 particles from 900 CCD frames. Iterative reconstruction on the most populated set corresponding to the DNA-bound form with 41 500 particles was continued until convergence was achieved using a final angular step of 3°. No symmetry was applied during the refinement. The map resolution was assessed based on the same method as for holoenzyme and final reconstruction was filtered to the estimated resolution of 23 Å calculated at FSC_0.5_ (18.5 Å using FSC_0.143_). Model fitting using crystal structures of DNA gyrase domains is described in Supplementary Methods. The cryo-EM maps of the holoenzyme and DNA-bound enzyme have been deposited in the EMDataBank under accession numbers EMD:2360 and EMD:2361.

## RESULTS

### DNA gyrase complex formation and analysis by supramolecular native mass spectrometry

Bacterial Topo2A enzymes share a high degree of primary sequence homology that extends to the structural level. To help overcome the technical barrier imposed by the flexible modular heterotrameric organization of the enzyme for structural studies, we worked with the thermostable bacteria *T. thermophilus* GyrA and GyrB orthologs. Apart from the *E. coli*-specific TOPRIM domain extension, the *T. thermophilus* subunits share ∼38 % identity with *E. coli* GyrA and 41% identity with *E. coli* GyrB (51% when excluding the TOPRIM insertion specific to *E. coli*).

We have performed a DNA supercoiling assay at 65°C and 37°C, the known optimal growth temperatures for *T. thermophilus* and *E. coli*, respectively (Figure 1B). The *T. thermophilus* enzyme is able to introduce supercoils but to a lesser extent than the *E. coli* DNA gyrase whose specific insertions have been shown to stimulate supercoiling activity (39,40). To prevent subunit dissociation and further stabilize the heterotetramer for structural studies, we used a construct (GyrBA-fus) containing both full-length GyrB and GyrA subunits via a linker connecting their C- and N-termini, respectively. The GyrBA-fus DNA gyrase is a catalytically active topoisomerase (Supplementary Figure S1A), which is able to supercoil DNA *in vitro* to the same level as the reconstituted heterotetramer complex. This result is in agreement with previous functional studies showing that similar fusion protein from TopoIV or DNA gyrase retain full activity and are suitable for crystallographic and biophysical studies (15,16,40–44).

To monitor complex formation, we used native mass spectrometry that preserves the oligomeric state of macromolecular assemblies in gas phase (45). Native mass spectrometry experiments confirmed the dimeric structure of the GyrBA-fus protein with a measured molecular mass of 320 377 ± 30 Da. In presence of ADPNP, a non-hydrolysable analog of ATP, the molecular mass of the dimer increases to 321 794 ± 51 Da, showing the addition of two ADPNP molecules per GyrBA-fus dimer, representing ∼0.3% of the total mass when compared with the GyrBA-fus dimer without nucleotide (320 377 ± 30 Da; Figure 1C and Supplementary Figure S2A).

To form the DNA-bound complex, a 155 bp DNA template was designed based on previous DNA footprinting studies (28–30). Native mass spectrometry analysis showed that addition of the 155 bp DNA template leads to the formation of a DNA-bound complex with a measured mass corresponding to one DNA template per GyrBA-fus dimer. Addition of ADPNP to the DNA-bound complex shows the presence of two nucleotide molecules (Figure 1C and Supplementary Figure S2B).

### Single-particle cryo-EM of the Holoenzyme and DNA-bound complex

To maximize the stability of a homogeneous complex, the GyrBA-fus protein was incubated with ADPNP to form the holoenzyme complex and cross-linked using glutaraldehyde. MALDI mass spectrometry analysis showed that cross-linking does not affect the dimeric conformation observed by native mass spectrometry (Supplementary Figure S3A). Trypsin digestion experiments coupled to MALDI mass spectrometry show that the same regions are prone to protease digestion in cross-linked and non–cross-linked samples indicating that the trapped dimer conformation is preserved in our experiment (Supplementary Figure S3B).

**Figure 2.**
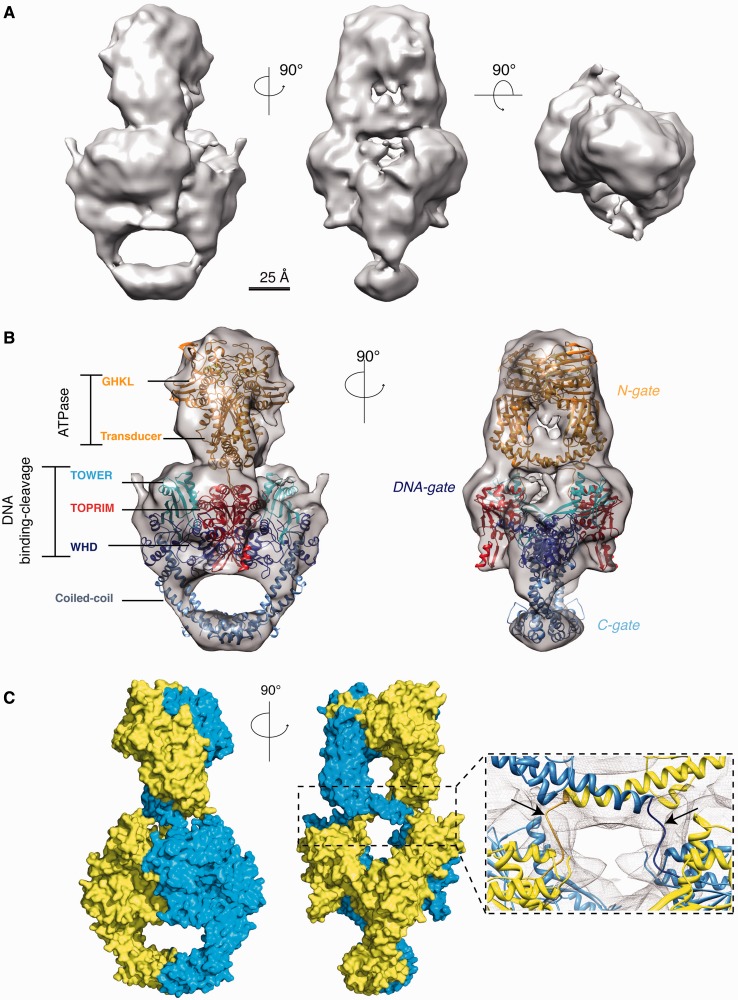
Orthogonal orientation and intertwined organization of the holoenzyme. (**A**) Cryo-EM map of the holoenzyme shown in three orientations. The cavities of the core GyrA and the ATPase domains appear clearly in the first two orientations in agreement with the crystal structures of these domains. (**B**) Model of the holoenzyme complex. The crystal structures of the ATPase domain (PDB:1EI1) and DNA binding–cleavage domain (PDB: 3NUH) deleted from the *E. coli* GyrB specific insertion domain [560–735] were fitted in the cryo-EM map (gray surface). The ATPase domain sits above DNA binding–cleavage domain in an orthogonal orientation. (**C**) Surface representation of the DNA gyrase full-length architecture and close-up of the DNA cavity interface fitted in the cryo-EM map of the holoenzyme (*gray mesh*). The orthogonal orientation of the two subunits leads to an intertwined dimer where one ATPase transducer helix connects to the TOPRIM domain on the opposite side through a 10 amino-acids linker (*pointed by the arrows*).

A data set of 62 322 molecular images was collected from frozen hydrated samples and was analyzed using single particle methods to reconstruct a 3D model from the most populated subset of 20 500 particles corresponding to the holoenzyme. The final refinement of the image orientation parameters was performed by projection matching and yielded a structure of the holoenzyme at ∼16.9 Å resolution by using the 0.5 Fourier Shell cutoff criteria (Figure 2A and Supplementary Figure S4A).

**Figure 3.**
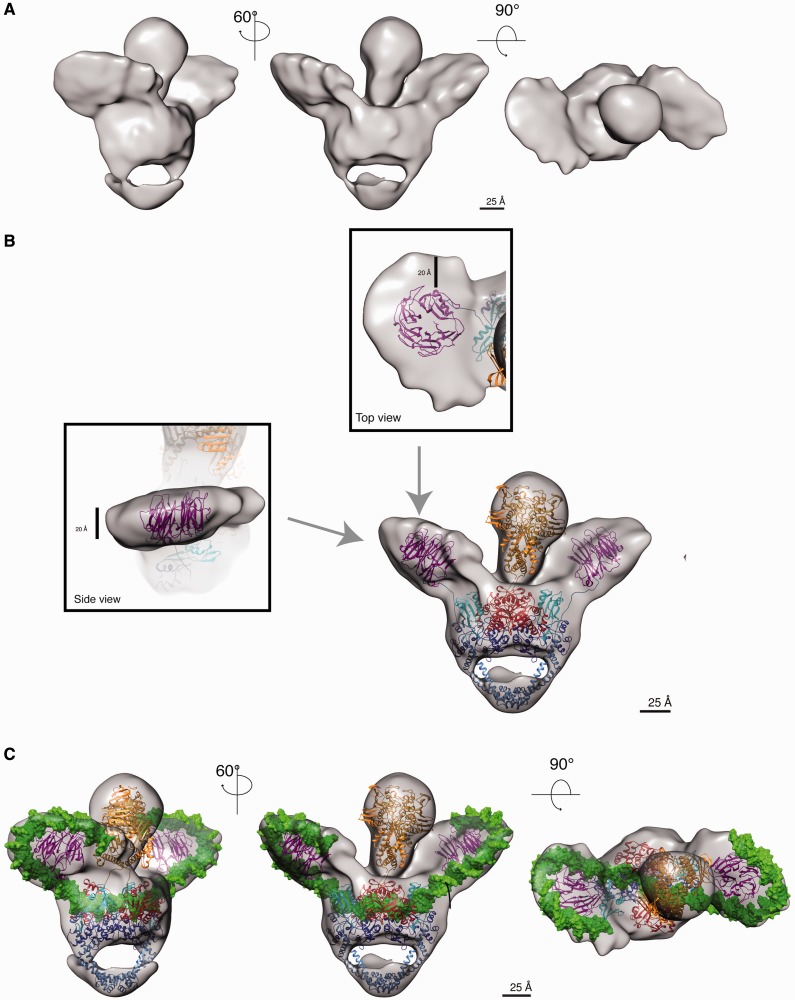
DNA stabilizes and wraps around DNA gyrase β-pinwheel CTDs. (**A**) Cryo-EM map of the DNA-bound complex shown in three orientations. Outside the core enzyme, the DNA-bound complex shows two additional disk-shape electron densities. (**B**) Fitting of the crystal structures of DNA gyrase domains in the cryo-EM map. The crystal structures of the ATPase (PDB:1EI1) and DNA binding–cleavage domain in presence of ciprofloxacin (PDB:2XCT) were fitted in the core enzyme map. The two additional densities on both side of the core enzyme can accommodate a β-pinwheel structure (PDB:3L6V), typical of DNA gyrase CTDs. The close-up of one CTD ß-pinwheel fitted in the cryo-EM map (*gray surface*) shows some empty extra densities large enough to position a dsDNA helix as visible on the top and side views. (**C**) Modeled duplex DNA wrapping around DNA gyrase. Extra densities around the β-pinwheels are large enough to accommodate DNA (*green*) that wraps around the two asymmetric β-pinwheels and binds in the DNA-gate cavity.

DNA gyrase is the target of the fluoroquinolone drugs that are potent broad-spectrum antibiotics. They stabilize the cleavage complex by intercalation of two molecules in the DNA binding–cleavage sites 4 bp apart, preventing DNA religation (15,16,18). We formed a ternary complex with the fluoroquinolone antibiotic ciprofloxacin to block the GyrBA-fus protein on a DNA sequence centered on a ciprofloxacin sensitive region and incubated with ADPNP to form the DNA-bound complex. We used the refined cryo-EM reconstruction of the holoenzyme as an initial model to generate a preliminary map of the DNA-bound complex out of a subset of ∼24 000 particles images that displayed additional densities when compared with the holoenzyme. The map of the DNA-bound complex was then iteratively refined with a subset of 41 500 particles coming from a larger data set by projection matching yielding a structure of the DNA-bound complex with a 0.5 Fourier-shell cutoff resolution of ∼23 Å (Figure 3A and Supplementary Figure S4B).

### Holoenzyme and DNA-bound complex model fitting

The holoenzyme map is composed of multiple domains that form two internal cavities consistent with the crystallographic structures of the ATPase and DNA binding–cleavage domains (40,46). The crystal structures of individual conserved domains of the highly homologous *E. coli* DNA gyrase could be fitted unambiguously into the holoenzyme map (Figure 2B). The resulting model reveals a linear N- to C- arrangement of the conserved domains and that the ATPase domain sits in an orthogonal orientation above the DNA binding–cleavage domain (∼105°) (Supplementary Figure S5A). This orientation brings the C-terminal end of the ATPase domain transducer helices at a 22 Å distance from the TOPRIM N-terminal end, which is compatible with the positioning of the 10 amino-acids connection between these domains in the electronic density (Figure 2C and Supplementary Figure S7A). The continuous electron density in this region allowed us to trace the linkers between the C-terminal end of the ATPase domain and the N-terminus of the TOPRIM domain crossing above the DNA-binding cavity, which is empty in the holoenzyme. As a consequence, the two subunits are forming an intertwined dimer similar to the one deduced from the recently reported crystal structure of the yeast Topo2A (Figure 2C) (14).

The crystal structures of *E. coli* ATPase domain (46) and of the *Staphylococcus aureus* DNA binding–cleavage domain with DNA and ciprofloxacin (16) could be fitted in our 3D reconstruction of the DNA-bound enzyme, revealing a similar orthogonal orientation of the ATPase domain on the DNA gate (Figure 3B and C), implying that the braided structure is a general structural feature when the dimer is formed in presence of ATP. The orientation of the ATPase domain with respect to the DNA binding–cleavage domain is ∼15°–25° different in the yeast structure from the one observed in our holo- and DNA-bound prokaryotic Topo2A, respectively, suggesting rotation movements of the ATPase domain around the dimer vertical axis (Supplementary Figure S5).

In the 3D reconstruction of the holoenzyme, no clear density can be observed at the expected positions of the CTDs along the enzyme core domains, suggesting that they are flexible in the absence of DNA. Protease digestion experiments coupled to MALDI mass spectrometry confirmed that these domains are highly accessible to proteolysis even in the cross-linked complex (Supplementary Figure S3B). Previous biophysical studies had shown that the CTDs could adopt multiple conformational states in solution (31,32). Symmetric densities budding on both sides of the enzyme catalytic domains appear at lower density threshold values, indicating the presence of a mixture of conformations that have most likely been averaged during the refinement process (Supplementary Figure S4C).

**Figure 4.**
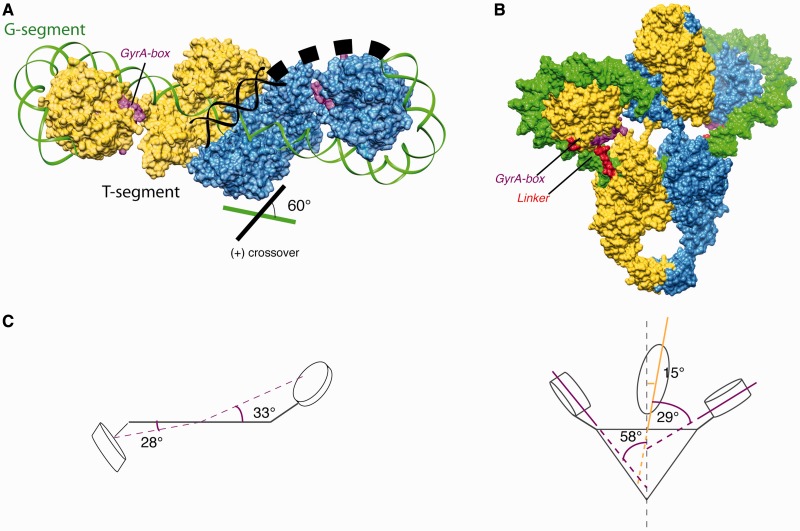
DNA path and domain geometry in DNA-bound complex. (**A**) Surface representation of the DNA binding–cleavage domain (top view). The ATPase domain has been omitted for clarity. The modeled 130 bp dsDNA is chirally wrapped around the CTD orienting the T-segment (*black*) in the DNA-gate groove formed by the TOPRIM-WHD and TOWER domains to form a 60° angle positive crossover when we extrapolate the T-segment path. The GyrA-box motif, colored in magenta, is facing the central cavity of the DNA binding–cleavage domain. (**B**) The conserved orthogonal orientation of the two monomers in the DNA-bound gyrase complex leads to an intertwined dimer with the ATPase domain sitting ∼10 Å higher on the DNA binding–cleavage domain (*surface representation in yellow and blue*). The linker (*red*) connecting the DNA binding–cleavage domain and the β-pinwheels domain is running along the modeled DNA (*green*), while the first blade bearing the GyrA-box motif (*magenta*) is facing the central DNA cavity. (**C**) Schematic representation of the domains orientation in the DNA-bound complex model. The DNA binding–cleavage domain (DNA- and C-gate) is depicted as a triangle, the GyrB ATPase domain (N-gate) as an ellipse, and the CTD β-pinwheel domains as disks. Magenta and orange solid and dash lines indicate the CTD and ATPase domain planes, respectively. (*Left*) The black horizontal line represents the DNA binding–cleavage plane in a top view. The CTDs β-pinwheels are located in an upper position and are distributed asymmetrically on each side of the DNA gate (33° versus 28°). (*Right*) The ATPase domain bends toward one CTD by an angle of ∼15°with respect to the vertical axis.

Strikingly, extra densities on both sides of the catalytic domains can be observed in the 3D reconstruction of the DNA-bound complex, compatible with the disk-like shape of the β-pinwheels structure (Figure 3A and B). Each β-pinwheel resides in an upper position, on the same side as its corresponding ATPase subunit. In our model for the DNA-bound complex, we positioned the linkers connecting the C-gate coiled-coil to the β-pinwheel along the GyrA tower domain as seen in the TopoIV ParC subunit crystal structure, the paralogous bacterial Topo2A (23). To avoid potential clashes with the ATPase domain during the catalytic cycle, we chose to orient the pinwheel such as the linker and the pinwheel C-terminus tail are pointing out from the outside surface of the dimer. As a result, the pinwheel blades are oriented such as the GyrA-box motif is facing the central DNA-binding cavity below the ATPase domain (Figure 4A and B).

The volumes deduced from the β-pinwheel structure are not sufficient to fill the densities that are large enough to accommodate a 20 Å-wide DNA double helix (Figure 3B). About 130 bp of a DNA helix could be modeled out of the initial 155 bp duplex centered in the DNA-gate groove and wrapping around the β-pinwheels (Figure 3C). The DNA is stretching out on both sides of the central cavity of the DNA binding–cleavage domain with a ∼35° angle on each side with respect to the central part of the DNA blocked by ciprofloxacin. In the DNA-bound map, the ATPase domain is ∼10 Å higher above the DNA-gate when compared with the holoenzyme map, indicating vertical movements of this domain on DNA binding. The ATPase domain is bent ∼15° toward one of the CTD β-pinwheel when compared with the holoenzyme map, suggesting a spatial proximity between these two domains during the catalytic cycle (Figure 4C).

### Requirement of the ATPase domain and CTD β-pinwheels in DNA gyrase activities

Most functional investigations on the role of the ATPase domain have been performed using point mutations or ATP analogs (47,48). To probe the structural contribution of the overall ATPase domain in DNA gyrase mechanisms, we performed DNA relaxation and supercoiling experiments of DNA gyrase completely lacking the ATPase domain (residues 1–392).

Some studies have reported that DNA gyrase is able to relax DNA in an ATP-independent manner *in vitro* (49). In our experiments, the ATPase deletion enzyme displays an ATP-independent DNA relaxation activity and is more efficient than the full-length enzyme (Supplementary Figure S1B). This shows that the ATPase domain is not required to form a functional DNA binding–cleavage domain, in contrast with the DNA gyrase CTD β-pinwheels whose deletion abolishes ATP-independent DNA relaxation (25).

Interestingly, the ATPase deletion enzyme shows a partial DNA supercoiling activity (Supplementary Figure S1C). In contrast with the full-length wild-type enzyme that generates negative supercoils in presence of ATP, our DNA supercoiling experiments show that the ATPase deletion enzyme introduces positive supercoils in relaxed DNA (Supplementary Figure S1C). DNA cleavage/religation is part of the enzymatic steps leading to supercoiling and is unaffected by the deletion of the ATPase domain. Consequently, this experiment suggests that the presence of the ATPase domain is essential in the first steps of the cycle, before DNA cleavage, and may be involved in the DNA crossover formation leading to negative supercoils.

In absence of ATP, the wild-type enzyme behaves like the ATPase deletion enzyme and shows a partial positive DNA supercoiling activity (Supplementary Figure S1D). Taken together these experiments suggest that closure of the ATPase domain is required for guiding DNA toward negative DNA supercoiling.

## DISCUSSION

### DNA path and role of the GyrA-box in the stabilization of the DNA crossover

Our cryo-EM data reveals for the first time the full architecture of DNA gyrase in absence and in presence of a 155 bp long DNA template, a structural state closer to the physiological context than previously observed in truncated enzyme with 20–34 bp DNA sequences. The comparison of our models for the holoenzyme and DNA-bound enzyme shows that the domain interface in presence of DNA defines a cavity large enough to accommodate two superposed DNA helices. In our observed conformation, the DNA path of our linear 155 bp DNA designed based on DNA footprinting studies is not long enough to loop back in the central cavity of the enzyme. However, the projected path of the DNA suggests that the β-pinwheels position the T-segment toward the N-gate entry to form an angle of 60° with the G-segment, compatible with the proposed geometry of positive DNA crossover in the DNA-gate leading to negative supercoiling after strand passage (Figure 4A and 4C) (20,23).

Furthermore, our structural data on the DNA gyrase show an asymmetric disposition of the CTD β-pinwheel domains, which are both stabilized by the presence of DNA (Figure 3C). The positions of the β-pinwheel domains induce a smaller ∼70° DNA bend angle outside the cleavage domain than previously reported in crystal structures of DNA binding–cleavage domains with 34 bp oligonucleotides (∼150°) (6,16). In our intact protein, the DNA gyrase CTD pinwheels direct the DNA outside the DNA binding–cleavage domain, preventing the DNA ends from moving upward as observed for CTD-less constructs in crystallographic studies. Such a small bend angle (∼75°) could also be observed for the TopoIV, whose CTD displays a partial pinwheel fold. In contrast, the reported mean bend angle measured in FRET and AFM studies are within the range of 150° for full-length eukaryotic Topo2A enzymes, with a CTD sharing no structural homology with the bacterial Topo2A (50). This may imply that the DNA bend angle might be determined by each Topo2A-specific CTD structure.

Complete DNA binding around the pinwheel surface generates an additional torsion of ∼220°–240° consistent with the reported wrapping around DNA gyrase pinwheel blades (51). If we extrapolate the T-segment trajectory based on the DNA-bound complex, we find that the conserved ‘GyrA-box’ would face the intersection of the DNA crossover (Figure 4A and B).

Most mutations affecting positively charged or aromatic residues along the β-pinwheel surface have deleterious effect on supercoiling and also affect DNA relaxation (52). Altogether, this suggests that the function of the ‘GyrA-box’ would not only be to structurally stabilize a circular shape that enforces DNA wrapping, but may also stabilize the crossover itself. This structural feature could also explain that DNA gyrase is able to perform ATP-independent relaxation of negative supercoiled DNA *in vitro* in contrast with all other Topo2A enzymes (49). With a complete deletion of the ATPase domain as shown in our experiments or in absence of ATP resulting in an open N-gate, the DNA crossover may still be stabilized enough for DNA relaxation, thanks to the GyrA-box position. During distributive ATP-independent relaxation of negative supercoil, the strong basic electrostatic potential of the circular β-pinwheel surface of DNA gyrase could bend the negative crossover and induce the two segments to cross such that they can fit in the DNA binding–cleavage site (Supplementary Figure S6A). In fact, the bacterial TopoIV enzyme, which lacks a full β-pinwheel CTD structure, fails to perform either ATP-independent relaxation or DNA supercoiling (23), reminiscent of the eukaryotic type II topoisomerases with divergent CTDs (2).

In addition, this β-pinwheel position implies that the enzyme C-terminal end faces outside the catalytic core domain. This orientation could accommodate species-specific gyrase C-terminal sequences avoiding steric hindrance with the ATPase domain and would allow specific contact with regulatory extensions in the TOPRIM domain as hypothesized for the *E. coli* enzyme (39,40) (Supplementary Figure S6B).

### The CTD and ATPase domain structurally cooperate to trap DNA crossovers during negative DNA supercoiling

Sequential ATP hydrolysis during the catalytic cycle provides the energy necessary to trigger conformational changes responsible for DNA translocation across the enzyme (9,53). That catalytic process is viewed as the ‘motor’ of the enzyme. Still, the precise role of the ATPase domain has long been debated (54). The structural relationship with the enzyme’s other domains during the catalytic cycle still has not been established in a definite fashion.

Recent FRET studies have pointed to a potential role of the CTD GyrA-box in contributing to inter-domain communication in DNA gyrase (41). From a structural standpoint, the spatial proximity of the ATPase domain and the CTD pinwheel in our conformation in the DNA-bound complex suggests that the two domains are able to structurally cooperate to capture the T-segment to form a positive crossover in the early steps of the catalytic cycle. The tilted position of the ATPase domain after ATP binding may reflect a short-lived intermediate that facilitates DNA capture.

Previous studies have demonstrated that plasmid DNA can wrap freely around isolated CTD generating a positive superhelicity imposed by the spiral-shaped β-pinwheels (24,26,51). The full-length DNA gyrase in absence of ATP as well as an ATPase deletion mutant as shown in our biochemical data are able to introduce a positive writhe in DNA via free wrapping around its specific DNA gyrase CTD pinwheel structure. Still, both constructs cannot maintain a positive crossover in the DNA binding–cleavage cavity stable enough to achieve negative supercoiling. This indicates that the ATPase domain is involved actively—not only in DNA capture but also in the structural stabilization of the crossover.

**Figure 5.**
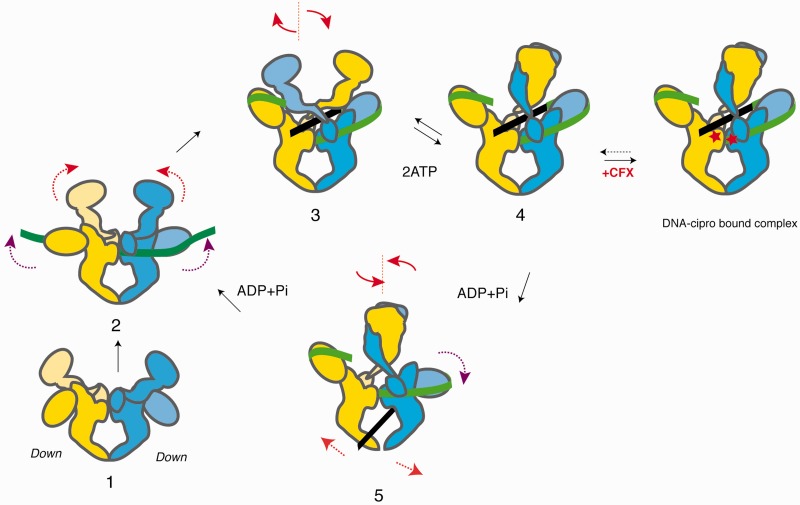
DNA gyrase supercoiling catalytic cycle in the light of DNA gyrase full architecture. (1) Before DNA binding, the ATPase domain is in a wide-open conformation and the CTDs are located in a lower position. (2, 3) Upon G-segment binding (*in green*), the DNA gyrase CTDs are rising up asymmetrically as one contiguous T-segment (*black*) wraps around the CTDs, narrowing and rotating the ATPase subunits in an upper-close position. The tilted position of the ATPase domain toward a CTD on one side may favor T-segment capture. (3, 4) ATP binding closes the ATPase domain dimeric interface and traps the T-segment to form a positive crossover with the G-segment. In this scheme, the swiveling ‘crossover-trap’ mechanism for T-segment capture could be common for all Topo2A. In this context, our structural data suggest that the ciprofloxacin antibiotic (red stars) could trap a T-segment pre-transport intermediate conformation according to the respective positions of the CTD and ATPase domain. (4, 5) The T-segment is guided from the N- to the C-gate by a lowering movement of the CTD domains through the DNA gate orthogonal opening. The G-segment is resealed after DNA gate closure promoting an efficient passage through the enzyme down to the C-gate. The second ATP is hydrolyzed to reset the enzyme for another strand passage. Extrapolation of the domains positions during the catalytic cycle is reminiscent of a ‘crawl-like’ coordinated swimming movement with sequential DNA wrapping guided by oscillations of the ATPase domain (*head*), T-segment transport supported by the β-pinwheel movements (*arms*) and orthogonal opening of the C-gate (*legs*).

The crossing of the ATPase domain subunits implies that on T-segment capture and ATP binding, the subunits may swivel up from an open position to a close braided dimer, locking down the T- and G-segments positive crossover in the DNA-gate cavity in a ‘crossover trap’ mechanism (Figure 5). As a consequence, the DNA crossover would be maintained under the positively charged surface of the ATPase domain C-terminal helices right before strand passage (Supplementary Figure S7B).

The eukaryotic Topo2A perform ATP-dependent relaxation of supercoils (2). In the conformation present in the yeast structure the ATPase domain C-terminal helices do not cross completely and form a smaller interaction network than the one observed in DNA gyrase, but still leading to an intertwined dimer. The strong sequence and structural conservation of the catalytic domain of the eukaryotic DNA topoisomerases (5) suggest that this ‘crossover trap’ mechanism might be a universal feature among all Topo2A. In contrast, the complete structure of a type 2B enzyme, the archeal TopoVI, shows that its transducer domain is limited to a single α-helice that cannot cross to form intertwined subunits. As a consequence, the TopoVI ATPase and DNA binding–cleavage domains display a planar organization that is not compatible with a crossover trap mechanism (55,56).

### DNA gyrase conformations during DNA strand passage

The fact that the Topo2A can perform a sequential strand passage is supported by all the biochemical data showing the ability to accommodate only one T-segment per cycle (47,48). SAXS and FRET analysis have shown that the β-pinwheels can be positioned at different vertical locations along the core enzyme (31,32). Our model of the enzyme bound to a 155 bp long DNA highlights the fact that these β-pinwheel domains can occupy asymmetric locations with respect to the body of the holoenzyme, helping with DNA capture and translocation. This asymmetric organization in horizontal and vertical planes suggests a ‘crawl-like’ movement where one β-pinwheel at a time guided by the ATPase domain positions the T-segment in the active site to achieve DNA supercoiling (Figure 5).

The quaternary organization of the ATPase and DNA binding–cleavage domains is a conserved feature between the yeast and bacterial Topo2A in presence of nucleotide. However, the ATPase dimer position versus the DNA binding domain displays different angles highlighting a significant range of rotational movements of the N-gate dimer that could be coordinated to distinct openings steps of the DNA and C-gates during the catalytic cycle. Electron densities at the N- and the C-gates are less defined in the DNA-bound state than for the holoenzyme, suggesting that the DNA-bound map reflects intermediate opening states of the C-gate in presence of DNA.

### Ciprofloxacin blocks a T-segment pre-transport conformation

The recent crystal structure of the yeast Topo2A has highlighted the role of the K-loop, a conserved ATPase domain motif with charged residues thought to promote the second ATP hydrolysis step that resets the enzyme (14). In this particular conformation, the K loops contact the ends of a 30 bp DNA duplex mimicking the conformation of a G-segment after T-segment transfer. In our bacterial gyrase/DNA complex model, the K loop is not positioned to contact the bent G-segment, and therefore represents a distinct conformational state.

The closed dimeric state of the ATPase domain observed in the cryo-EM map of our DNA-bound complex is indicating that ciprofloxacin was able to block DNA in the enzyme DNA gate cavity, in presence of the non-hydrolysable analog of ATP. This observation is in agreement with a previous mutagenesis study of the homologous *Streptococcus pneumoniae* TopoIV that showed the appearance of ciprofloxacin-resistant mutations of conserved residues involved in the ATPase dimerization and outside of the quinolone binding region, indicating that ciprofloxacin targets an ATP-bound state of the bacterial Topo2A (57).

Moreover, crystal structures of the central DNA binding domain of type 2 A topoisomerases have shown a variety of open and closed conformations of the C-gate that are correlated to the formation of the covalent bond between the active tyrosine and the DNA during the phosphate backbone cleavage (7,14). Sequential gate openings are regulated such as the release of the T-segment cannot occur before DNA religation to prevent the accumulation of lethal double strand breaks (54). The quinolone molecules interfere with DNA religation by intercalating in the cleavage sites and act as ‘poisons’, blocking the cleavage complex. The crystal structures of DNA gyrase cleavage complex in presence of a small DNA and quinolones restricted to the DNA binding–cleavage domain have shown that the C-gate is closed, as it also appears in the 3D reconstruction of our DNA-bound complex with ciprofloxacin. Together with the CTD pinwheel upper position displayed in our map which is compatible with a T-segment capture during the early stages of the catalytic cycle, we can hypothesize that the observed conformation of our DNA-bound complex is thus more likely to represent a T–segment pre-transport state blocked by ciprofloxacin (Figure 5) (16,18). When most of the knowledge accumulated on the DNA topoisomerase–drug complexes have been focusing on the atomic details of the drug interaction with their target site, our result highlights the fact that cryo-EM structures, although not providing atomic details, are able to reveal the enzyme conformations targeted by drugs and could be useful information to better target-specific steps of the catalytic cycle.

In conclusion, this study has revealed for the first time the domain connections and organization of a full-length bacterial Topo2A as well as the DNA path leading to negative supercoiling by DNA gyrase. Our data provides a new insight into the structural cooperation of the ATPase and CTDs throughout the catalytic cycle. Further analysis of DNA gyrase complexes with different DNA substrates or drugs will provide snapshots of the multiple conformations that DNA gyrase can adopt during the catalytic cycle.

## ACCESSION NUMBERS

EMD:2360, EMD:2361.

## SUPPLEMENTARY DATA

Supplementary Data are available at NAR Online: Supplementary Figures 1–7, Supplementary Methods, Supplementary Movies 8–9 and Supplementary References [58–63].

## FUNDING

Marie Curie International Reintegration Grant from the European Community FP7 program; doctoral grant from the Alsace Region and CNRS; the Fondation pour la Recherche Médicale; Instruct, part of the European Strategy Forum on Research Infrastructures (ESFRI) supported by national member subscriptions as well as the French Infrastructure for Integrated Structural Biology (FRISBI) [ANR-10-INSB-05-01]. Funding for open access charge: Fondation ARC pour la Recherche sur le Cancer.

*Conflict of interest statement.* None declared.

## Supplementary Material

Supplementary Data
