# Supplementary material for: Structural insight into negative DNA supercoiling by DNA gyrase, a bacterial type 2A DNA topoisomerase
Source: Nucleic Acids Res. 2013 Jun 26;41(16):7815–27. doi: 10.1093/nar/gkt560 (PMC3763546; doi:10.1093/nar/gkt560)
Supplement: Supplementary Data [file supp_41_16_7815__index.html]

Structural insight into negative DNA supercoiling by DNA gyrase, a bacterial type 2A DNA topoisomerase — Structural insight into negative DNA supercoiling by DNA gyrase, a bacterial type 2A DNA topoisomerase — Supplementary Data 

# Structural insight into negative DNA supercoiling by DNA gyrase, a bacterial type 2A DNA topoisomerase

## 

files

**Files in this Data Supplement:**

- Supplementary Data - pdf file
- Supplementary Data - mp4 file
- Supplementary Data - mp4 file
