# Supplementary material for: Structural insight into negative DNA supercoiling by DNA gyrase, a bacterial type 2A DNA topoisomerase
Source: Nucleic Acids Res. 2013 Jun 26;41(16):7815–27. doi: 10.1093/nar/gkt560 (PMC3763546; doi:10.1093/nar/gkt560)
Supplement: Supplementary Data [file supp_gkt560_nar-00323-m-2013-File007.pdf]

SUPPLEMENTARY INFORMATION : Papillon *et al.*

SUPPLEMENTARY METHODS

Wild type *and mutant T.thermophilus* and *E.coli* DNA gyrase cloning

The coding regions for full-length *Thermus thermophilus* HB8 strain GyrA (1-805) and GyrB (1-634) and *Escherichia coli* K12 strain GyrA (1-875) and GyrB (1-804) were amplified from genomic DNA. Both *T.thermophilus* GyrB and GyrA subunits were cloned into the NdeI and BamHI restriction sites of a pET30a vector containing a N-terminal hexahistidine (His6) tag upstream of a Thrombine protease cleavage site (GE Healthcare Life Science). Both *E.coli* GyrB and GyrA subunits were cloned into the NdeI and BamHI restriction sites of an in-house modified pET28a vector containing a N-terminal decahistidine (His10) tag upstream of a PreScission protease cleavage site (P3C, GE Healthcare Life Science). The *T.thermophilus* construct of the ATPase deletion mutant [2- 392] of GyrB (GyrBΔN)was generated using restriction enzymes and cloned into the same pET28a-derived vector.

Protein expression and purification

Wild type and mutant T. thermophilus DNA Gyrase subunits

Protein expression was carried out in *E.coli* BL21 (DE3) cells. One-Liter cultures were grown in LB-media in 2 l flasks at 37°C to an optical density (A600 nm) of 0.6-0.8. Protein expression was induced by the addition of 0.7 mM isopropyl-b-D- thiogalactopyranoside (IPTG) for 3H at 37°C. 15g of BL21(DE3) cells were resuspended in lysis buffer (Tris-HCl 50mM pH8, KCl 300mM, PMSF 0.1mM, Protease Inhibitor Cocktail (cOmplete, EDTA-free) tablets (Roche), Glycerol 5%) and sonicated. After centrifugation, the supernatant was loaded on a Ni2+-affinity column (GE Healthcare Life Science). The column was washed with buffer A300 (Tris-HCl 50mM pH8, KCl 300mM). The protein was then eluted with buffer B300 (buffer A300 containing 250 mM imidazole), and digested with protease. The sample was dialyzed overnight at 4°C in a 14,000 molecular weight cut-off (MWCO) dialyzing membrane against buffer TD-100 (Tris-HCl pH8 50mM, 100nM KCl, DTT 0.1mM) complemented by 2mM CaCl2. The dialyzed sample was then adsorbed to an anion exchange column (HiTrap HQ, GE Healthcare Life Science) equilibrated in buffer TD-100 and eluted using a 100– 600 mM KCl gradient

over 20 column volumes. Peak fractions were concentrated and run over a size exclusion column (Sephadex S200 16/60, GE Healthcare Life Science) equilibrated in buffer TD-100.

Wild type E.coli DNA Gyrase B

Protein expression was carried out in *E.coli* BL21 (DE3) cells. One-Liter cultures were grown in LB-media in 2 l flasks at 37°C to an optical density (A600 nm) of 0.6-0.8. Cultures were transferred to 18°C and protein expression was induced by the addition of 0.35 mM isopropyl-b-D-thiogalactopyranoside (IPTG) for 16H at 18°C. 15g of BL21(DE3) cells were resuspended in lysis buffer (Hepes 20mM pH8, NaCl 800mM, ß-mercaptoethanol 1mM, Imidazole 30 mM, PMSF 0.1mM, PIC tablets, Glycerol 10%) and sonicated. After centrifugation, the supernatant was loaded on a Ni2+-affinity column (GE Healthcare Life Science). The column was washed with buffer A50 (Hepes 20mM pH8, NaCl 50mM, ß-mercaptoethanol 1mM, Imidazole 30 mM, Glycerol 10%). The protein was then eluted with buffer B50 (buffer A50 containing 250 mM imidazole), and digested with protease. The sample was dialyzed overnight at 4°C in a 14,000 molecular weight cut-off (MWCO) dialyzing membrane against buffer C50 (Hepes 20mM pH8, NaCl 50mM, Glycerol 10%, EDTA 1mM, DTT 1mM). The dialysed sample was then adsorbed to an anion exchange column (HiTrap HQ, GE Healthcare Life Science) equilibrated in buffer C50 and eluted using a 50– 500 mM NaCl gradient over 20 column volumes. Peak fractions were concentrated and run over a size exclusion column (Sephadex S200 16/60, GE Healthcare Life Science) equilibrated in buffer D500 (Hepes 20mM pH8, KCl 500 mM, Glycerol 10%, EDTA 1mM, DTT 1mM).

Wild type E.coli DNA Gyrase A

Protein expression was carried out in *E.coli* BL21 (DE3) cells. One-Liter cultures were grown in LB-media in 2 l flasks at 37°C to an optical density (A600 nm) of 0.6-0.8. Protein expression was induced by the addition of 0.35 mM isopropyl-b-D- thiogalactopyranoside (IPTG) for 4H at 37°C.

6g of BL21(DE3) cells were resuspended in lysis buffer (Hepes 20mM pH8, NaCl 1000mM, ß-mercaptoethanol 1mM, Imidazole 20 mM, PMSF 0.1mM, PIC tablets, Glycerol 10%) and sonicated. After centrifugation, the supernatant was loaded on a Ni2+-affinity column (GE Healthcare Life Science). The column was washed with buffer A100 (Hepes 20mM pH8, NaCl 100mM, ß-mercaptoethanol 1mM, Imidazole 20 mM, Glycerol 10%). The protein was then eluted with buffer B100 (buffer A100 containing 250 mM imidazole), and digested with protease. The sample was dialyzed overnight at 4°C in a 14,000 molecular weight cut-off (MWCO) dialyzing membrane against buffer C100 (Hepes 20mM pH8, NaCl 100mM, ß-mercaptoethanol 1mM, Glycerol 10%). The dialyzed sample was concentrated and run over a size exclusion column (Sephadex S200 16/60, GE Healthcare Life Science) equilibrated in buffer D400 (Hepes 50mM pH8, KCl 400 mM, Glycerol 10%, EDTA 1mM, DTT 1mM).

Fusion T. thermophilus DNA gyrase

Protein expression was carried out in *E.coli* BL21 (DE3) cells. One-Liter cultures were grown in LB-media in 2 l flasks at 37°C to an optical density (A600 nm) of 0.4-0.6. Cultures were transferred to 18°C and protein expression was induced by the addition of 0.8 mM isopropyl-b-D-thiogalactopyranoside (IPTG) for 16H. 15g of BL21(DE3) cells were resuspended in lysis buffer (Hepes 20mM pH8, NaCl 500mM, MgCl2 5mM, DTT 1mM, imidazole 10 mM, PMSF 0.1mM, PIC tablets, Glycerol 5%) and sonicated. After centrifugation, the supernatant was loaded on a Ni2+-affinity column (GE Healthcare Life Science). The column was washed with buffer A500 (Hepes 20mM pH8, NaCl 500mM, MgCl2 5mM, DTT 1mM, 10 mM imidazole, Glycerol 5%) followed by buffer A1000 (Buffer A500 but with 1M NaCl). The protein was then eluted with buffer B1000 (buffer A1000 containing 250 mM imidazole), and digested with P3C protease. The sample was dialyzed overnight at 4°C in a 14,000 molecular weight cut-off (MWCO) dialyzing membrane against buffer TGED-80 (Tris-HCl pH8 10mM, Glycerol 10%, EDTA pH8 1mM, DTT 1mM, NaCl 80 mM). The dialyzed sample was then adsorbed to an anion exchange column (HiTrap HQ, GE Healthcare Life Science)

equilibrated in buffer TGED-80 and eluted using a 80– 1000 mM NaCl gradient over 20 column volumes. Peak fractions were concentrated and run over a size exclusion column (Sephadex S200 16/60, GE Healthcare Life Science) equilibrated in buffer TGED-500 (TGED buffer with 500 mM NaCl).

DNA preparation

The 155-bp DNA sequence from the pBR322 plasmid covering a major quinolone sensitive site at position 990 which included positions 915-1064 was inserted in 16 copies in tandem repeat into the pCRBlunt vector (Invitrogen) at a EcoRV restriction site, similar to a procedure described by Dyer *et al*. (pCR-149-16) (58). The pCR-149-16 vector was prepared using plasmid Giga prep kit (Qiagen). Each 155bp were extracted by enzymatic digestion and purified by gel filtration (Tris 50 mM pH8, NaCl 150mM) on a Sephadex S200 10/30 column (GE Healthcare Life Science). Fractions were pooled and dialyzed overnight against water.

Mass spectrometry

Instrument parameters optimization for native mass spectrometry

In order to transfer the intact protein complexes ions, it was not necessary to cool the ions by increasing the pressure in the first vacuum stage of the mass spectrometer. This could be related to the interface configuration of the Bruker instrument as compared to Waters instruments where a pressure of about 5-7mbar is necessary to detect multimers complexes ions (45) with m/z>5000. Calibration was performed by using a concentrated solution of CsI (1mg/ml in water:isopropanol 1:1). Masses were calculated and averaged for all ions of the charge state distribution. The difference between experimental and theoretical mass measurement is consistent with the fact that incomplete removal of solvent and buffer molecules is often observed when ESI-MS is performed on large protein complexes, causing an increase in mass as compared to the expected one (59).

Crosslinked Protein MALDI-TOF analysis:

The GyrBA-fus protein was pre-incubated with 0.5 mM ADPNP 20 min at 37°C and then mixed with 0.05% glutaraldehyde 2 min at 25°C in a buffer containing 20 mM Tris– acetate pH7.9, 100 mM potassium acetate, 10 mM magnesium acetate and 1mM DTT. The crosslinking reaction was stopped by the addition of 100 mM Tris-HCl pH8.0. The sample buffer was exchanged into 100 mM ammonium bicarbonate (pH7.8) using mini gel filtration columns (Microbiospin 30, Biorad). MALDI experiments were performed on an Autoflex II mass spectrometer (Bruker Daltonic, Bremen, Germany) equipped with a high mass detector HM1 (CovalX, Zurich, Switzerland). The samples were diluted to 4 µM and sinapinic acid was used as matrix. Calibration was performed using a mixture of standard proteins (PSII, Bruker). Non-crosslinked samples have been prepared under the same condition as a control.

Crosslinked Protein Limited proteolysis followed by MALDI mass spectrometry:

1µM of GyrBA-fus was pre-incubated with 0.5 mM ADPNP 20 min at 37°C and then mixed with 0.05% glutaraldehyde 2 min at 25°C in a buffer containing 20 mM Tris– acetate pH7.9, 100 mM potassium acetate, 10 mM magnesium acetate and 1mM DTT. The crosslinking reaction was stopped by the addition of 100 mM Tris-HCl pH8. The sample buffer was exchanged into 100 mM ammonium bicarbonate (pH7.8) using mini gel filtration columns (Microbiospin 30, Biorad). Non-crosslinked samples have been prepared under the same condition as a control. Samples were treated with Trypsin (Trypsin Gold MS grade, Promega) using a 1:200 ratio of Trypsin: protein at 37°C for different times. Samples were concentrated in a speedvac to 20µL. Samples were run on a 4-12% gradient Bis-Tris gel (Invitrogen), 1X MES-SDS at 200V for 35min. Gels were stained with colloidal blue (Invitrogen). SDS separated protein bands were excised from the gel and washed according the published protocol (60). The proteins were further reduced with 10mM DTT in 25 mM $AcONH_4$ for 45 min at 56°C and then alkylated with 55mM iodoacetamide in 25mM AcONH4 for 45min at room temperature. The proteins were then digested with trypsin and the digest peptides were extracted

with 35:65:5 eau:acetonitrile:formic acid solution. MALDI mass measurements were carried out on an Autoflex II (Bruker, Germany). Prespotted anchorchip targets (PAC system from Bruker Daltonik, technical note TN-011) with a-cyano-4-hydroxycinnamic acid (HCCA) matrix were used to analyse tryptic digests.

Biochemical assays

The reconstitution of DNA gyrase complexes was performed in relaxation buffer condition during 30 minutes at 37°C or 55°C for *E.coli* or *T.thermophilus* enzyme respectively. Negatively supercoiled plasmid DNA (pUC19) was purified using a maxiprep kit (Macherey–Nagel) and relaxed with DNA topoisomerase I prepared in-house from *E. Coli*.

DNA relaxation assays

Enzymes at varying concentrations were each incubated with 3.5nM negatively supercoiled DNA plasmid in 10-20 µl reactions for 30 min at 37°C. Assay conditions were 20 mM Tris-acetate pH7.9, 100 mM potassium acetate, 10 mM magnesium acetate, 1mM DTT. Samples were treated with 0.2% SDS and 0.5 mg/mL Proteinase K for 45 minutes at 55°C prior to electrophoresis. Samples were run on a 1% agarose, 0.5X TBE gel at 9V/cm for 3-4H. Gels were stained with 0.5mg/ml ethidium bromide (EtBr) in 0.5X TBE for 30min, followed by a brief destaining in 0.5X TBE. Bands were visualized using a Typhoon 8600 scanner.

DNA supercoiling assays in presence of ATP

Enzymes at varying concentrations were each incubated with 3.5nM relaxed DNA plasmid in 10-20 µl reactions for 30 min at 37°C. Assay conditions were 20 mM Tris-acetate pH7.9, 100 mM potassium acetate, 10 mM magnesium acetate, 1mM DTT and 1mM ATP. Samples were treated with 0.2% SDS and 0.5 mg/mL Proteinase K for 45 minutes at 55°C prior to electrophoresis. Samples were run on a 1% agarose, 0.5X TBE gel at 9V/cm for 3-4H. Gels were stained with 0.5mg/ml ethidium bromide (EtBr) in 0.5X TBE for 30min, followed by a brief destaining in 0.5X TBE. Bands were visualized using a Typhoon 8600 scanner.

SUPPLEMENTARY INFORMATION : Papillon *et al.*

DNA supercoiling assays in absence of ATP

These assays were performed in the same conditions as above with relaxed DNA except ATP was omitted for both the mutant enzyme devoid of ATPase domain and for the control wild type DNA gyrase. Samples were treated with 0.2% SDS and 0.5 mg/mL Proteinase K for 45 minutes at 55°C prior to electrophoresis.

Chloroquine two-dimensional electrophoresis (2D gels).

All samples for the 2D electrophoresis were treated with 0.2% SDS and 0.5 mg/mL Proteinase K for 45 minutes at 55°C prior to electrophoresis. Samples were run on a 1% agarose, 0.5X TBE gel in a first dimesion at 9V/cm for 4H. Gels were then incubated in a 0.5X TBE+ 2µg/mL chloroquine bath for 1H and run in a second dimension (90°) for 2H at 9V/cm in 0.5X TBE+ 2µg/mL chloroquine. Gels were stained with 0.5mg/ml ethidium bromide (EtBr) in 0.5X TBE for 30min, followed by a brief destaining in 0.5X TBE. Bands were visualized using a Typhoon 8600 scanner.

Sample preparation for cryo-EM Imaging

For the holoenzyme preparation, the purified GyrBA-fus was concentrated on a 2mL-vivaspin (Sartorius stedim, 10,000 MWCO PES) to a final concentration of 2mg/mL. The protein was then dialysed overnight against 20mM Hepes pH8, 100 mM NaCl, 5mM MgCl2, 1mM DTT. The sample was diluted to a final concentration of 150ng/µL in the same buffer and incubated with 0.5mM ADPNP for 15 min at 25°C. In order to stabilize the dimeric A_2B_2 complex, glutaraldehyde was added to a final concentration of 0.05% 20 s prior to vitrification. For the DNA-bound enzyme preparation, the previous purified and dialyzed GyrBA-fus was incubated with several ratio of 155bp DNA in presence of 0.5mM ciprofloxacin for 20 min at 37°C and then with 0.5 mM ADPNP before freezing without any crosslinking agent.

Two to three µL of the sample were deposited on QUANTIFOIL® R 2/2 holey grids subjected to a 10s air glow-discharge (0.1 mbar, 340V and 2.5mA). Vitrification was performed at 95% humidity, 10°C using a semi-automated device (Vitrobot,FEI).

SUPPLEMENTARY INFORMATION : Papillon *et al.*

Multiple alignements

Multiple alignments for DNA gyrase A (P0AES4, P056S3, Q07702, O33926, Q8DPM, P20831, QSSIL4, B7ASR3, O51396, O67108) and DNA gyrase B (P0AES6, P0S6S2, P0C5C5, P77993, P0A4M0, P0A0K8, Q5SHZ4, B7A9M1, P33769, O67137) were generated in Jalview using the Muscle webservice (http://www.compbio .dundee.ac.uk/jabaws).

Model fitting

Holoenzyme map

The PDB file 3NUH was modified to remove the *E.coli* specific insertion [560-735] in the TOPRIM domain to generate 3NUHΔinsert better matching the *T.thermophilus* enzyme domain organization. Crystal structures of DNA gyrase dimeric domains corresponding to the ATPase domain (1EI1) and DNA binding-cleavage domain (3NUHΔinsert) were fitted into the electron microscopy density maps using UCSF Chimera "fit in map" tool and adjusted manually (61). A correlation factor of 0.92 was calculated using a map simulated from the atoms coordinates at the expected resolution and allowing rotation and translation.

DNA-bound map

Crystal structures of DNA gyrase dimeric domains (ATPase domain:1EI0, DNA binding-cleavage domain:2XCT, CTD pinwheels:3L6V) were fitted into the electron microscopy density maps using UCSF Chimera "fit in map" tool and adjusted manually. A correlation factor of 0.88 was calculated using the map simulated from the atom coordinates at the expected resolution. Initially a 155bp DNA was modeled using the 3D-DART webserver (62) and positioned manually using COOT (63). After a global adjustment, we positioned about 130 bp in the map.

Figures

Molecular graphics images were produced using the UCSF Chimera package and Pymol (The PyMOL Molecular Graphics System, Version 1.5.0.1 Schrödinger, LLC.).

SUPPLEMENTARY REFERENCES

58. Dyer, P.N., Edayathumangalam, R.S., White, C.L., Bao, Y., Chakravarthy, S., Muthurajan, U.M. and Luger, K. (2004) Reconstitution of nucleosome core particles from recombinant histones and DNA. *Methods Enzymol*, 375, 23-44.

59. Bereszczak, J.Z., Barbu, I.M., Tan, M., Xia, M., Jiang, X., van Duijn, E. and Heck, A.J. (2012) Structure, stability and dynamics of norovirus P domain derived protein complexes studied by native mass spectrometry. *J Struct Biol*, **177**, 273-282.

60. Jensen, O.N., Wilm, M., Shevchenko, A. and Mann, M. (1999) Peptide sequencing of 2-DE gel-isolated proteins by nanoelectrospray tandem mass spectrometry. *Methods Mol Biol*, 112, 571-588.

61. Pettersen, E.F., Goddard, T.D., Huang, C.C., Couch, G.S., Greenblatt, D.M., Meng, E.C. and Ferrin, T.E. (2004) UCSF Chimera--a visualization system for exploratory research and analysis. *J Comput Chem*, **25**, 1605-1612.

62. van Dijk, M. and Bonvin, A.M. (2009) 3D-DART: a DNA structure modelling server. *Nucleic Acids Res*, **37**, W235-239.

63. Emsley, P. and Cowtan, K. (2004) Coot: model-building tools for molecular graphics. *Acta Crystallogr D Biol Crystallogr*, **60**, 2126-2132.

SUPPLEMENTARY INFORMATION : Papillon *et al.*

SUPPLEMENTARY FIGURES LEGENDS

Figure S1: DNA relaxation and supercoiling activity assays.

A. DNA supercoiling activity of *T.thermophilus* DNA gyrase fusion construct. Increasing concentration of *T.thermophilus* DNA gyrase fusion protein (GyrBA-fus) is incubated at 65°C with relaxed pUC19 plasmid in presence of ATP and run on an agarose gel colored with ethidium bromide. The enzyme fusion is active in the same concentration range as the reconstituted *T.thermophilus* DNA gyrase (A_2B_2) (see Figure 2A).

B. DNA relaxation assays of wild type and mutant DNA gyrase. Negatively supercoiled pUC19 plasmid was incubated with several concentrations of either reconstituted *T.thermophilus* DNA Gyrase A_2B_2 or reconstituted ATPase deletion mutant $A_2B_2\Delta N$ showing increasing DNA relaxation pattern.

C. DNA supercoiling assay of ATPase deletion mutant DNA gyrase. Relaxed pUC19 plasmid was incubated with increasing concentrations of reconstituted *T.thermophilus* DNA Gyrase $A_2B_2\Delta N$ showing a partial supercoiling pattern in absence of ATP.

On the right, the supercoiled DNA samples corresponding to the mutant DNA gyrase $A_2B_2\Delta N$ (300nM) in absence of ATP and to wild type DNA gyrase (0.23 nM) in presence of ATP were run on a two-dimensional chloroquine gels.

D. DNA supercoiling assay of wild type DNA gyrase in absence of ATP. Relaxed pUC19 plasmid was incubated with increasing concentrations of reconstituted *T.thermophilus* DNA Gyrase A_2B_2 showing a partial supercoiling pattern in absence of ATP.

On the right, the supercoiled DNA samples corresponding to DNA gyrase (300nM) in absence of ATP and to DNA gyrase (0.23 nM) in presence of ATP were run on a two-dimensional chloroquine gels.

N: nicked open circular DNA, R: relaxed DNA, SC: supercoiled DNA, +veT: positive topoisomers, -veT: negative topoisomers.

Figure S2: Native mass spectrometry of the holoenzyme and DNA-bound complexes.

A. Native mass spectrometry analysis of the DNA gyrase:ADPNP complex (holoenzyme). (a) Native gel analysis of the holo-or DNA-bound enzyme in presence or absence of ADPNP, a non-hydrolysable analogue of ATP. Addition of ADPNP to DNA gyrase produces a single shifted band in the gel indicating formation of the complex. Addition of DNA yields higher migrating species showing DNA binding in presence and absence of ADPNP. (b) ADPNP binding to DNA gyrase followed by native mass spectrometry. Mass spectra of the fusion DNA gyrase showed the only presence of the dimeric DNA gyrase fusion A_2B_2 in presence of ADPNP. Peaks are labeled with the charge state of the dimer. (c) The zoom capture shows the displacement of the mass spectra in presence of ADPNP. Voltage variation (250 – 400V) allowed the determination of the complex stoechiometry corresponding to 2 ADPNP molecules for one dimer protein as deduced from the measured molecular masses.

B. Native mass spectrometry analysis of the DNA gyrase:DNA:ADPNP complex (DNA-bound complex). (a) Determination of the stoechiometry of the DNA gyrase:DNA complex in presence of ADPPNP. The mass spectra of the fused DNA gyrase reveals only one oligomeric form, namely DNA gyrase:DNA:ADPNP with a 2:1:2 stoechiometry. Peaks are labeled with the charge state of the dimer. (c) The zoom capture shows the displacement of the mass spectra in presence of ADPNP.

Figure S3: Oligomeric conformation of the crosslinked holoenzyme.

A. MALDI mass spectrometry analysis of the crosslinked and non-crosslinked holoenzyme. (a) The non-crosslinked sample shows a 3-peaks pattern. The peaks correspond mainly to a monomeric fused DNA gyrase (163,828 Da) with a few dimeric species (323,202 Da). The last peak could correspond to a slight proteolysis of the fusion protein leading to two 90kDa sub-fragments. The crosslinked sample shows a 2-peaks pattern. They correspond mainly to the dimeric state (326,262 Da) stabilized by glutaraldehyde and a few monomeric species (165,831 Da). There is no non-specific interaction or aggregates and the sample is mainly dimeric in presence of ADPNP after glutaraldehyde crosslinking as expected.

B. Comparison of the proteolysis patterns of crosslinked and non-crosslinked holoenzyme samples. (a) SDS-gel analysis of the proteolysis pattern of the crosslinked and non-crosslinked samples. The samples were submitted to a 1-hour trypsin digestion. The arrows indicate the bands analysed by MALDI mass spectrometry. The profile is highly similar for the two samples. (b) Table represents the MALDI mass spectrometry analysis of the trypsin-digested bands. The colors characterize the different domains according to Figure 1A. The m/z column represents the peptide mass over charge ratio. The sequence column indicates the assigned peptide in the fused DNA gyrase sequence. The cross-mark indicates the presence of the peptides either in the non-crosslinked or the crosslinked samples. (c) Schematic representation of fused DNA gyrase proteolysis pattern on its sequence. Dark grey indicates fragments that are sensitive to trypsin proteolysis. The similar pattern of the managed proteolysis indicates that the glutaraldehyde does not affect and preserve the protein conformation in presence of ADPNP.

Figure S4: Image and cryoEM map analysis of DNA gyrase holoenzyme and DNA-bound complexes.

A. CCD images, Fourier Shell Correlation Function, class averages and re-projections of the holoenzyme complex. (a) CCD frame of the holoenzyme at 59,000x magnification on a Tecnai G2 Polara F30 cryo-electron microscope operated at 100 keV with an Eagle 4kx4k CCD camera (FEI) and close up of an isolated particle representative of the 3D-map conformation. (b) For the holoenzyme, the 0.5 FSC criterion gives a resolution of 16.9 Å and the Rosenthal and Henderson criterion (0.143 FSC) gives a resolution of 12.5 Å. (c**)** Raws 1-3-5 represent the class-averages and raws 2-4-6 are the corresponding 2-D reprojections of the final volume.

B. CCD images, Fourier Shell Correlation Function, class averages and re-projections of the holoenzyme and DNA-bound enzyme (a) CCD frame of the DNA-bound complex at 59,000x magnification on a Tecnai G2 Polara F30 cryo-electron microscope operated at 100 keV with an Eagle 4kx4k CCD camera (FEI) and close up of an isolated particle representative of the 3D-map conformation. (b) For the DNA-bound complex, the 0.5 FSC criterion gives a resolution of 23 Å and

the Rosenthal and Henderson criterion (0.143 FSC) gives a resolution of 18.5Å. (c) Raws 1-3-5 represent the class-averages and raws 2-4-6 are the corresponding 2-D reprojections of the final volume.

C. Representation of the cryo-EM map of the holoenzyme complex at different thresholds. The map at three different thresholds reveals some discrete densities along the central cavity (side view and top view) . These low densities at multiple positions pointed by arrows could correspond to the flexible CTD ß-pinwheel domains. These domains seem to be averaged during the refinement process due to their multiple conformations.

Figure S5: ATPase domain orientations in the yeast and *T. thermophilus* Topo2A complexes.

A. DNA gyrase organization in absence of DNA. The domains color-coding is the same as Figure 1A. On the left view with the upper part of the ATPase domain omitted, the ATPase domain transducer α-helices and the DNA binding-cleavage domain form a ~105°angle.

B. DNA gyrase organization in presence of a 155bp DNA. On the left view with the upper part of the ATPase domain omitted, the ATPase domain transducer α-helices and the DNA binding-cleavage domain form a ~95°angle.

C. Yeast Topo2A organization in presence of a 30bp DNA as seen in Schmidt et al. (Schmidt et al., 2012). On the left view with the upper part of the ATPase domain omitted, the ATPase domain transducer α-helices and the DNA binding-cleavage domain form an ~80°angle.

Figure S6: Interaction of the DNA crossover and the DNA gyrase insertion with the β-pinwheels.

A. Schematic representation of DNA gyrase and DNA TopoIV recognition of positive or negative crossovers. During ATP-dependent relaxation of positive supercoiled DNA, each DNA segment (G-or distal T-) is bound to the closed-rounded β-pinwheel of DNA gyrase to produce a positive crossover. The truncated β-pinwheel of the TopoIV can only contact one segment (distal T-) at a time and is sensing particularly positive nodes. During ATP-dependent DNA supercoiling, DNA gyrase can wrap a

contiguous T-segment (*dash lines*) to form a positive crossover. During ATP-independent relaxation of negative supercoiled DNA, it was hypothesized that the DNA gyrase β-pinwheel particular shape and positively charged midriff would attract both DNA segments (*black arrows*) to locally maintain a positive node.

B. Surface representation of the DNA-bound gyrase model showing the β-pinwheels orientation. DNA has been omitted from this view. The β-pinwheels have been positioned such as both the N-terminal and C-terminal (*red dots*) extremities point out of the dimer. The TOPRIM domain (red) of *E.coli* DNA gyrase (3NUH) was superposed on the TOPRIM domain of *S.aureus* (2XCT), revealing the position of the *E. coli*-specific TOPRIM domain insertion (red surface) in the context of the DNA-bound model. The CTD-tail extension of the *E.coli* enzyme is represented as magenta dash lines. This orientation could prevent steric hindrance with the ATPase domain but also promote extension-specific regulatory interaction as shown in recent studies (39).

Figure S7: ATPase – TOPRIM domain interface

A. Multiple alignments of eukaryotic Topo2A and prokaryotic DNA gyrase B centered on the region tethering the ATPase and the TOPRIM domain. The junction between the C-terminal end of the ATPase domain (1EI0) and the N-terminal end of the TOPRIM domain (3NUH-2XCT) is about 10 amino acids long. The secondary structure prediction in this region suggests that the RRK motif missing in the available crystal structures forms a final turn in the ATPase transducer α-helices and that the LPxxLA motif is starting a β-strand-like structure in the TOPRIM domain. This leaves only 3 to 7 amino acids to connect these 2 secondary structure elements, making it unlikely for the loop to fold back and connect in a non-intertwined manner.

B. Potential surface representation of the holoenzyme model. The potential surface was generated in Pymol with the APBS tool. Red to Blue represents negative to positive charged surface. The cross-sections at the DNA binding interface of the model show positively charged surfaces for both the DNA binding-cleavage domain cavity and the ATPase domain transducer α-helices surface (black

arrows-yellow dash line). This charge repartition is compatible with a mechanism where the G-segment is bound in the DNA-gate cavity and the T-segment maintained by the α-helices of the N-gate.

Figure S8: Movie of the holoenzyme cryoEM map and model fitting.

Figure S9:Movie of the DNA-bound cryoEM map and model fitting.

Figure S1

A. DNA supercoiling assays of fused *T.thermophilus* DNA gyrase

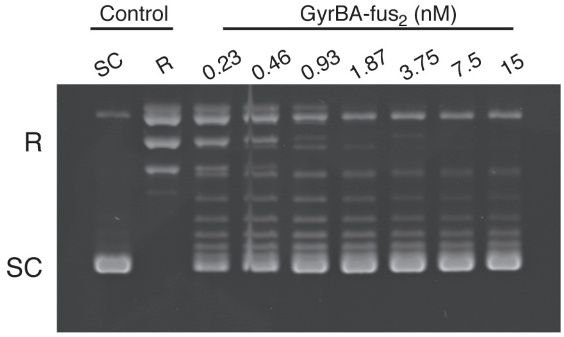

B. DNA relaxation assays of *T.thermophilus* DNA gyrase and ATPase deleted mutant

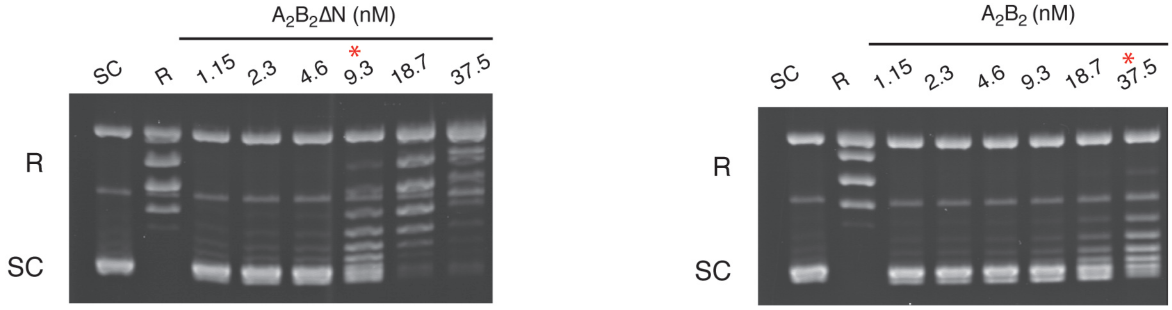

C. DNA supercoiling assays (-ATP) of *T.thermophilus* DNA gyrase ATPase deleted mutant and two-dimensional agarose gel

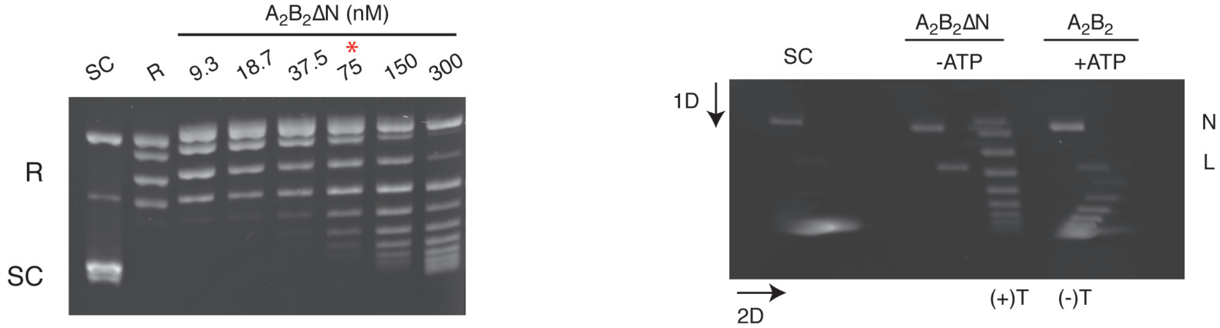

D. DNA supercoiling assays (-ATP) of *T.thermophilus* DNA gyrase and two-dimensional agarose gel

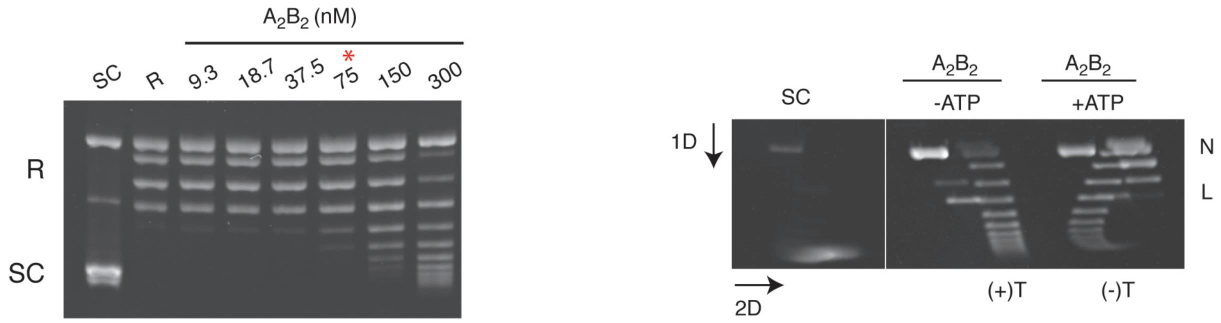

Figure S2

A.

(a)

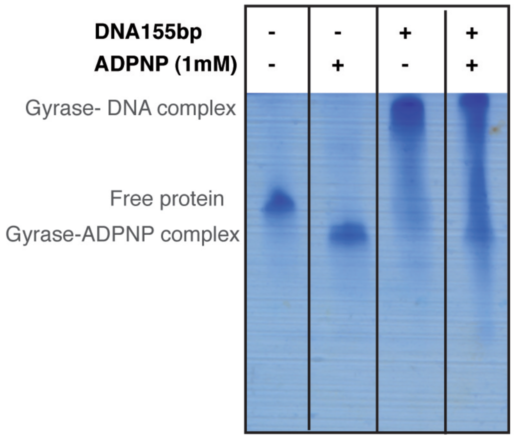

(b)

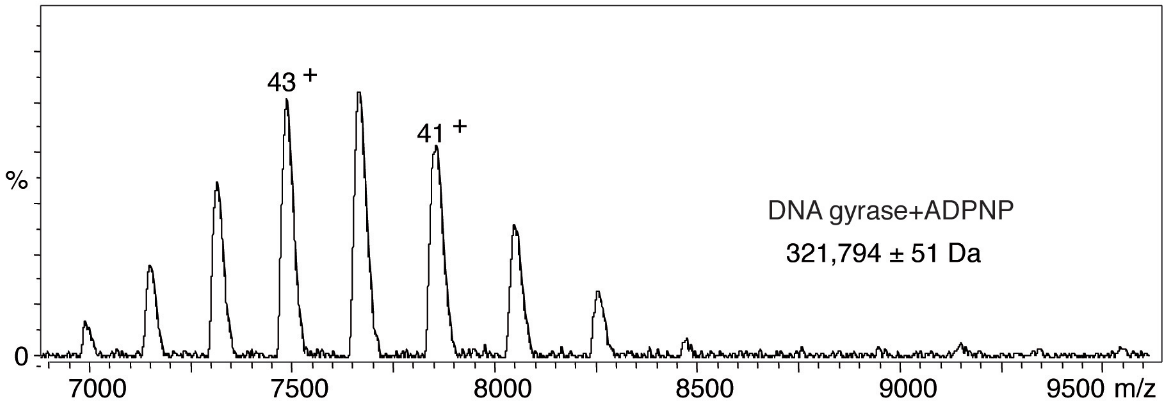

(c)

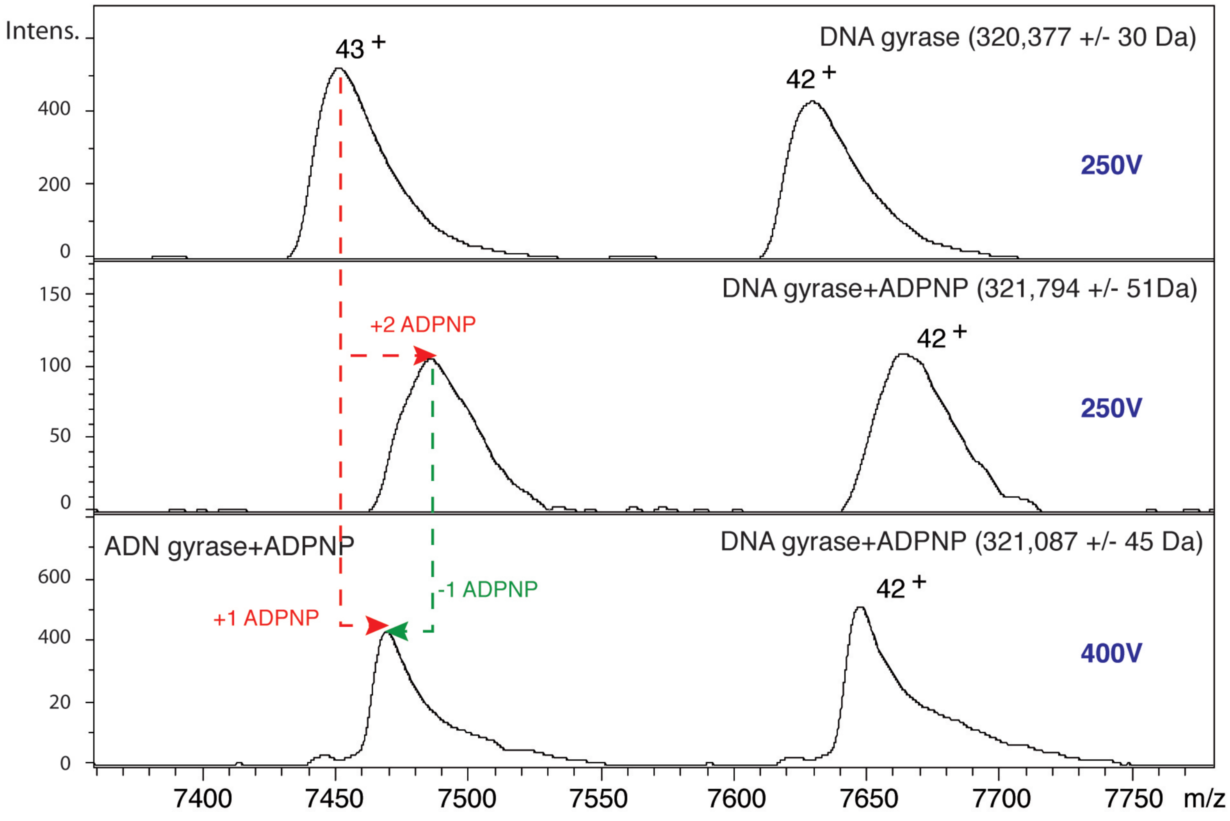

Figure S2

B.

(a)

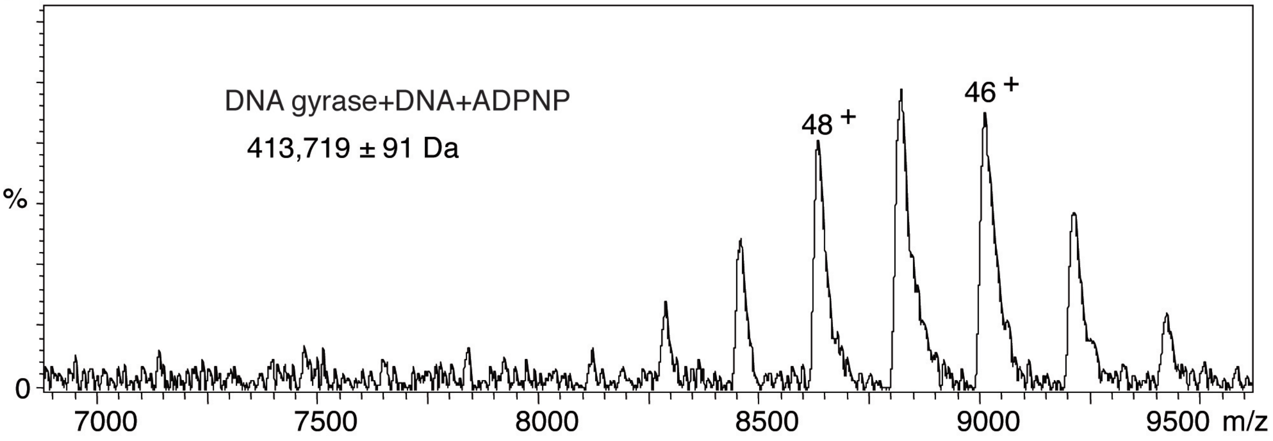

(b)

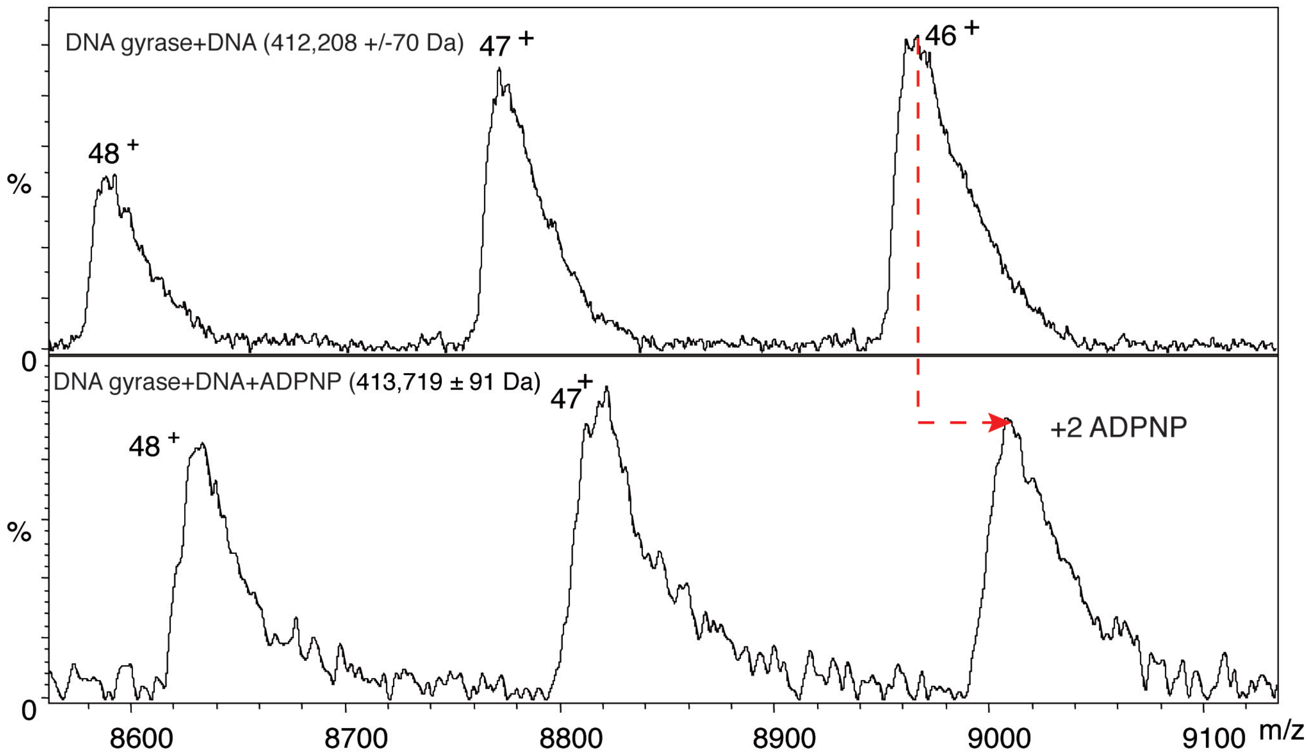

Figure S3

A.

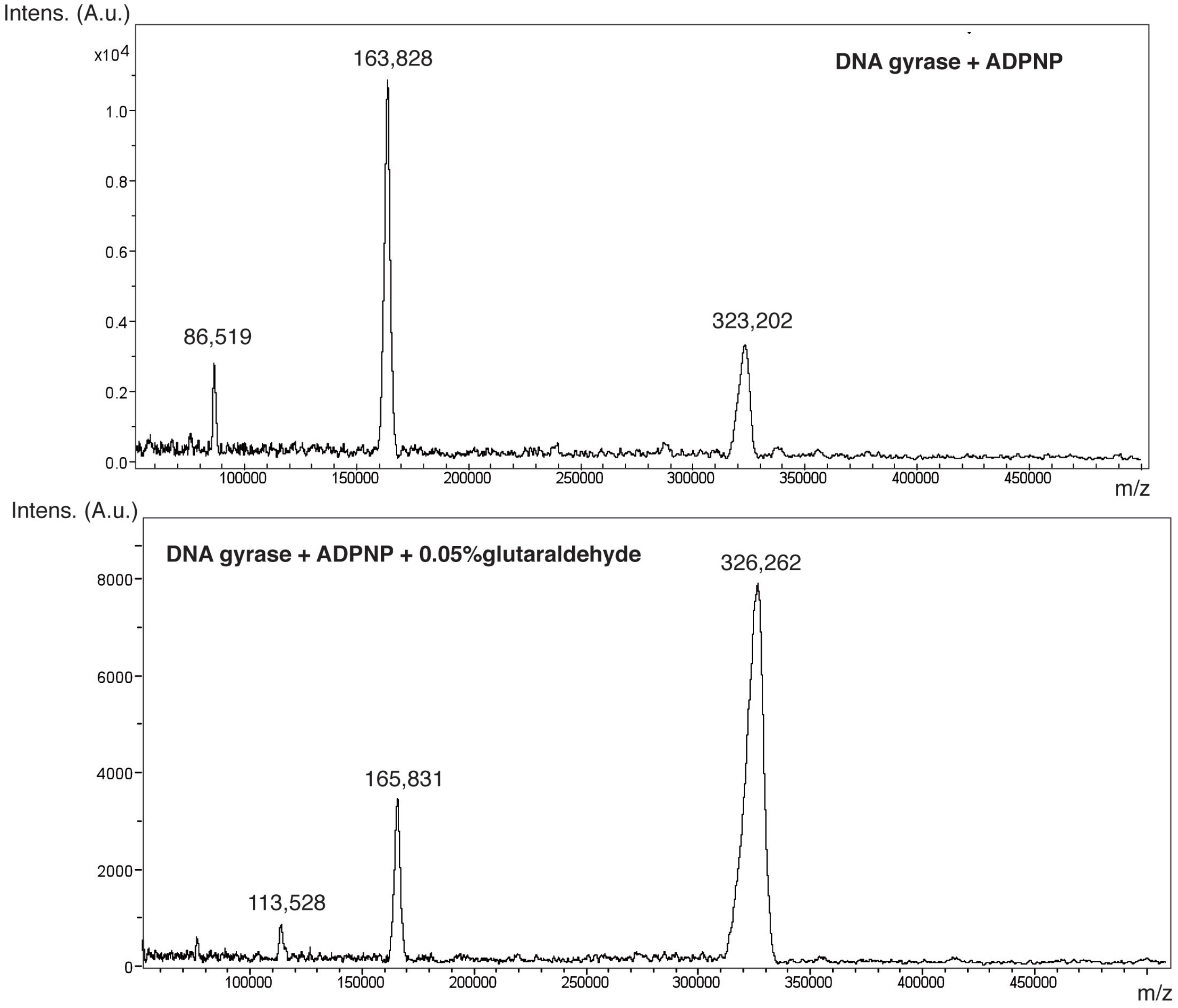
Intens. (A.u.)
x10^4
1.0
0.8
0.6
0.4
0.2
0.0
163,828
DNA gyrase + ADPNP
86,519
323,202
100000
150000
200000
250000
300000
350000
400000
450000
m/z
Intens. (A.u.)
DNA gyrase + ADPNP + 0.05%glutaraldehyde
326,262
8000
6000
4000
2000
0
165,831
113,528
100000
150000
200000
250000
300000
350000
400000
450000
m/z

Figure S3

B.

(a)

	-	+	Glutaraldehyde (0.05%)
	+	+	ADPNP (1mM)

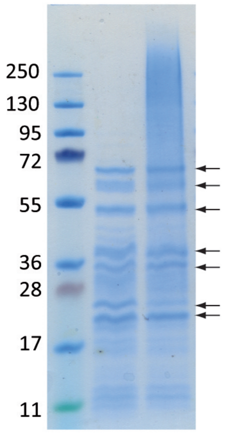

(b)

m/z	Sequence	Non-crosslinked sample	Crosslinked sample
1036,5	1-10	x	x
2270,1	20-39		x
2275,1	40-60	x	x
1518,7	61-74	x	x
2669,4	109-134	x	x
900,4	146-153	x	x
2233,2	161-180	x	x
1331,9	190-201	x	
1977	227-243	x	x
1816	339-355	x	x
1225,7	356-365	x	x
2371,1	468-491	x	
1655,8	495-509	x	x
1173,7	510-518	x	x
1077,6	519-526	x	x
1413,8	527-538	x	x
1614,8	545-557		x
831,3	565-570	x	x
1876,9	596-611	x	x
1000,5	612-620		x
1186,6	621-629		x

m/z	Sequence	Non-crosslinked sample	Crosslinked sample
1642,8	653-666		x
1647,9	682-696		x
1519,9	682-695	x	x
1697,9	711-725	x	x
1015,5	726-733		x
2288,1	734-755	x	x
1940,1	885-901	x	x
1197,7	902-913	x	
1042,6	916-924	x	x
1059,6	975-983	x	x
840,3	984-989		x
2423,4	1008-1029	x	x
1649,8	1045-1059	x	x
1033,6	1148-1155	x	x
2886,4	1169-1193	x	x
1093,5	1201-1209	x	x
1925	1218-1235	x	x
1597,8	1236-1249	x	x
1545,8	1259-1272	x	x
2495,2	1279-1302	x	x
889,4	1303-1309	x	x
1219,7	1314-1324	x	x
919,5	1323-1329	x	x
2227,2	1330-1350	x	
1416,8	1355-1366	x	x
1260,7	1356-1366	x	x
1313,8	1389-1400	x	
1058,5	1391-1400	x	x

(c)

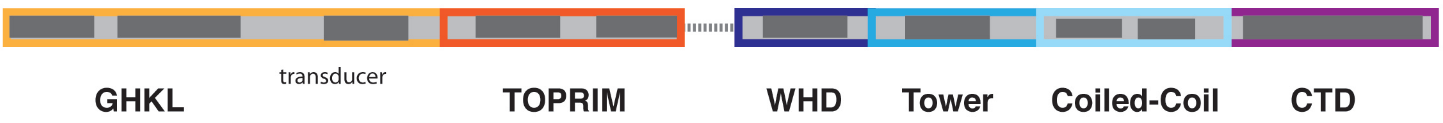

Figure S4

A. Holoenzyme

(a)

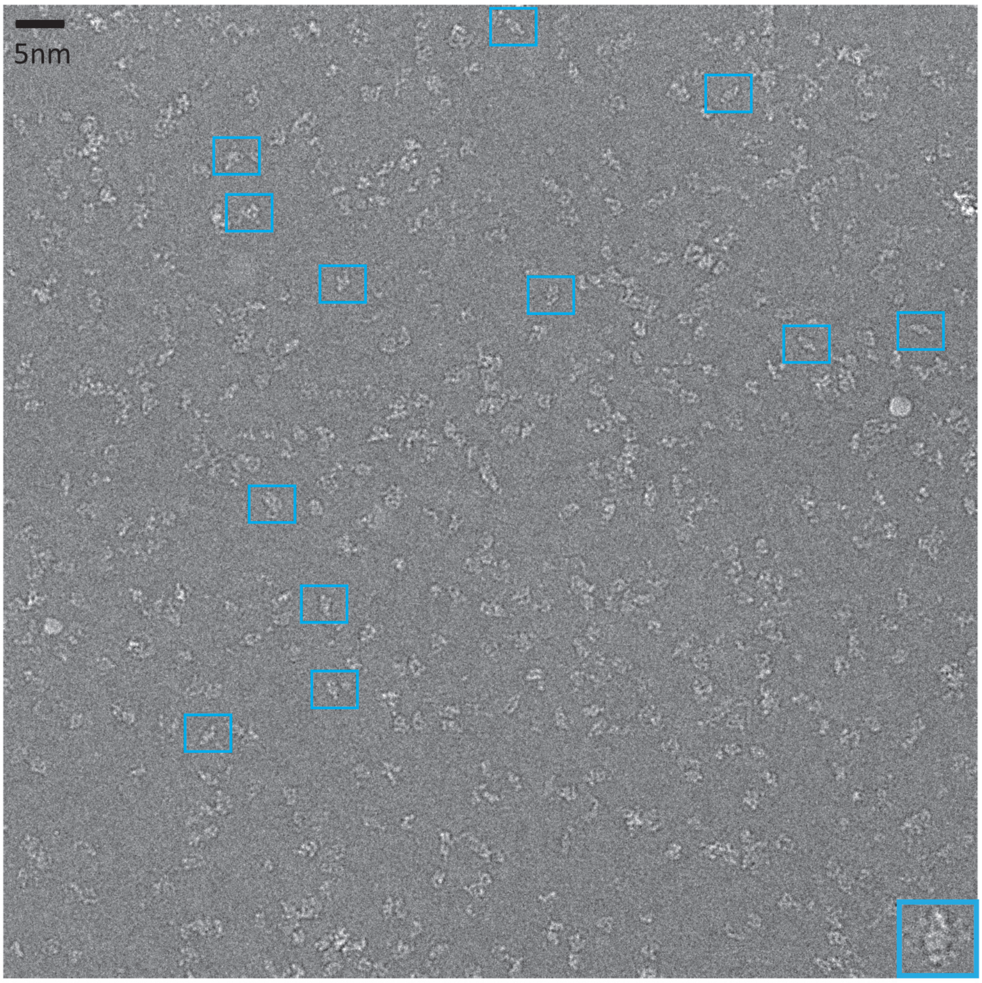

(b)

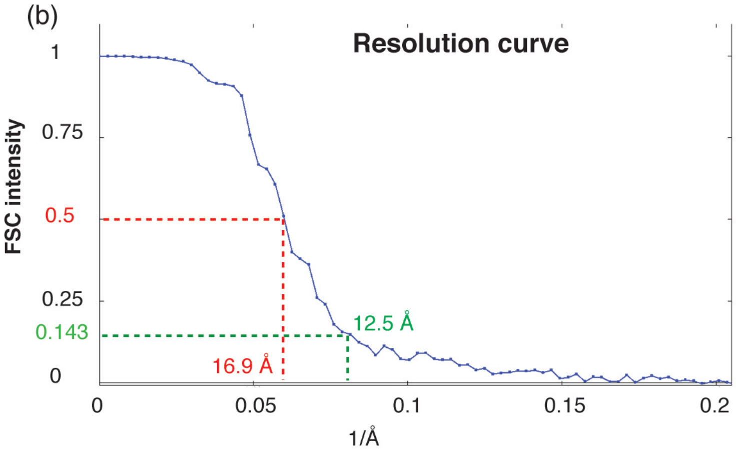

(c)

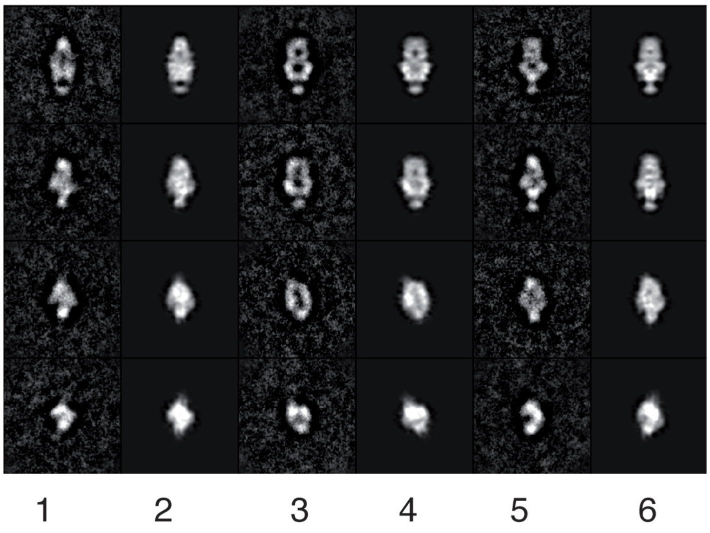

Figure S4

B. DNA-bound enzyme

(a)

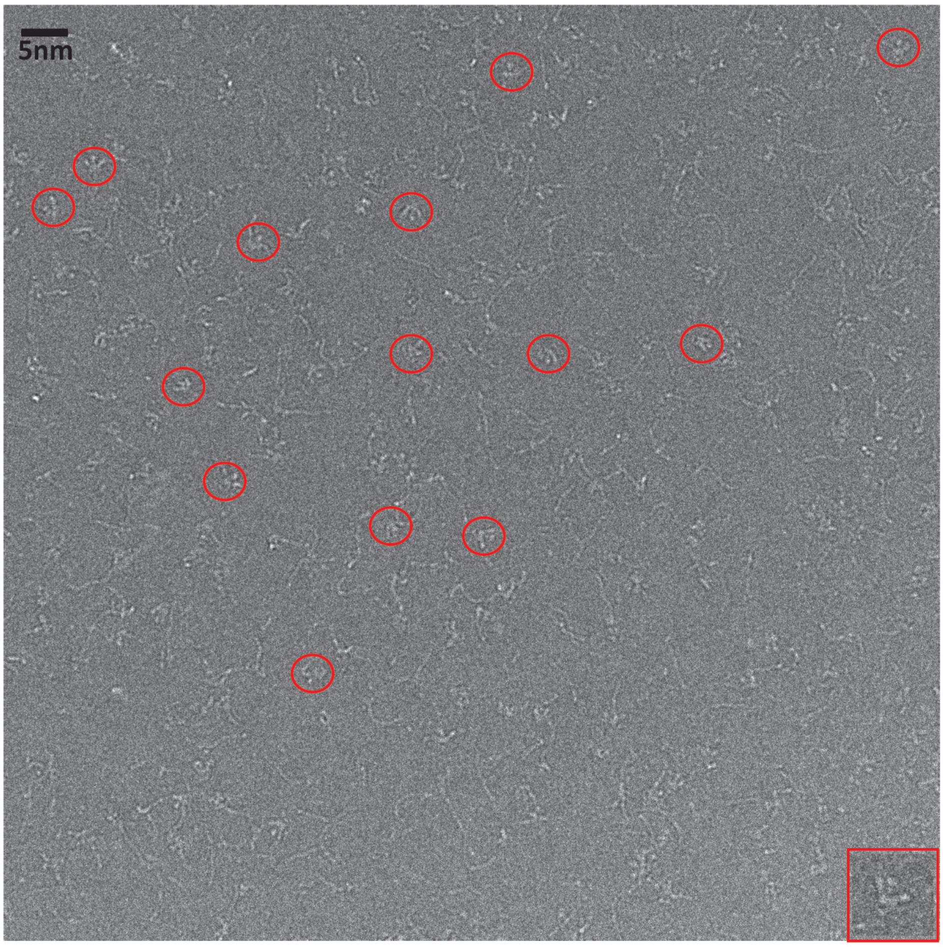

(b)

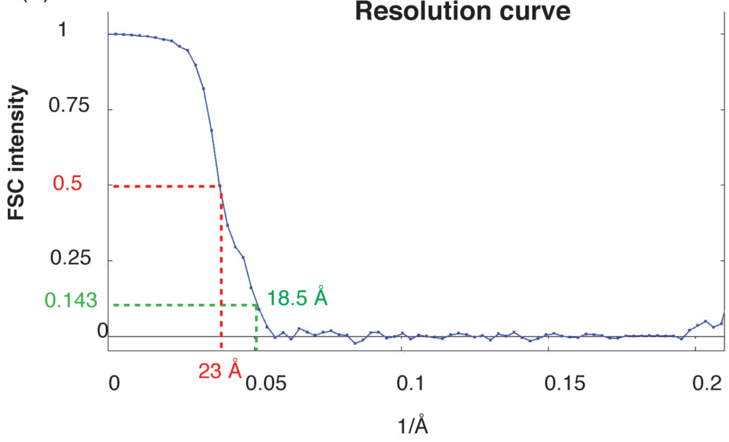

(c)

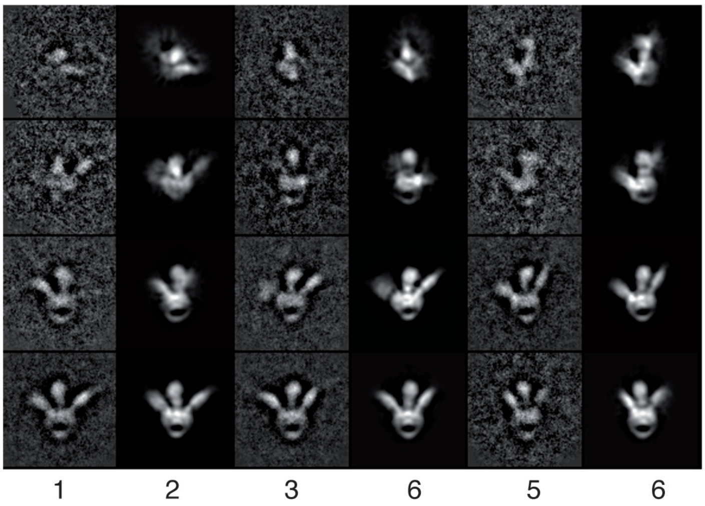

Figure S4

C.

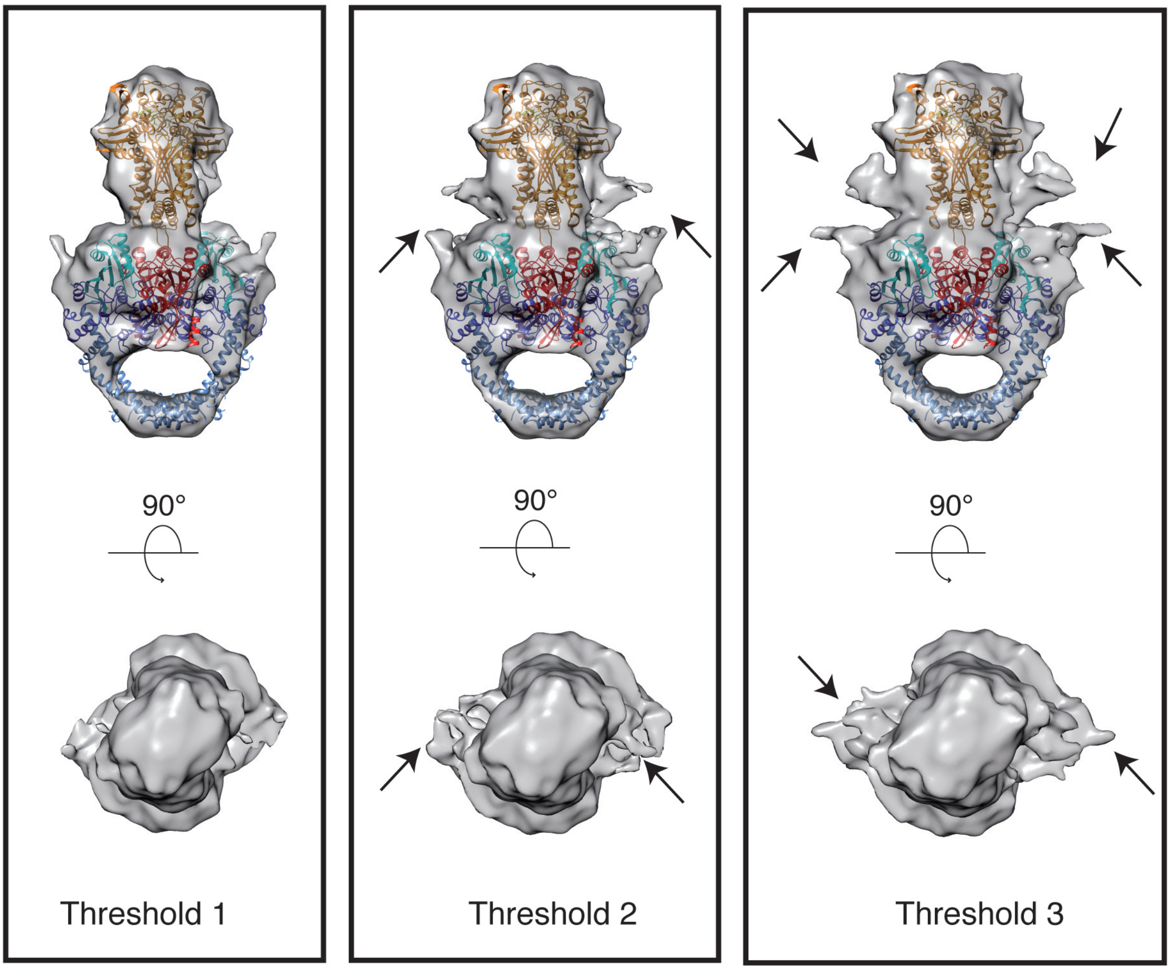

Figure S5

A. Holoenzyme complex of *T.thermophilus* DNA gyrase (+ ADPNP)

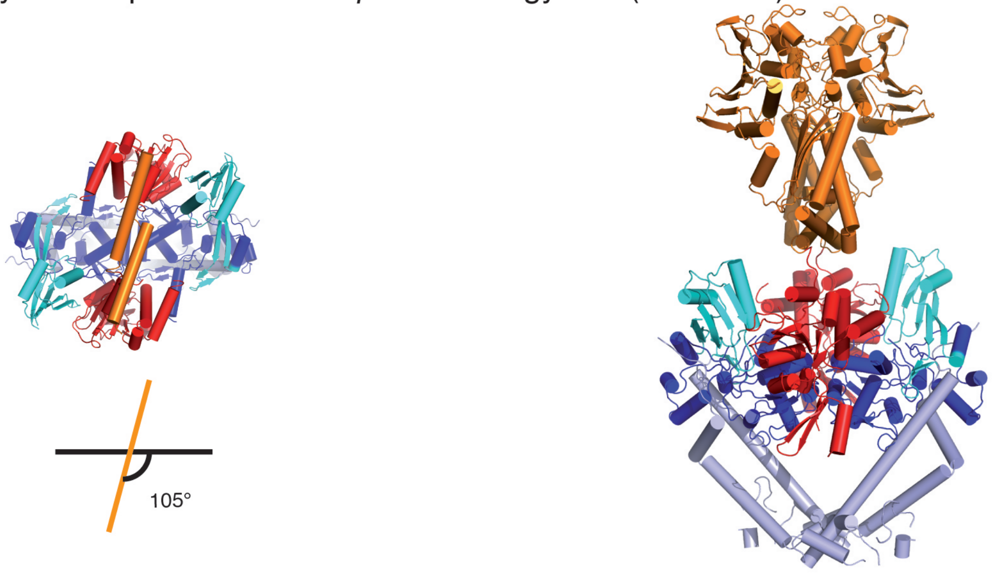

B. DNA-bound complex of *T.thermophilus* DNA gyrase (+ DNA 155bp + ADPNP + CFX)

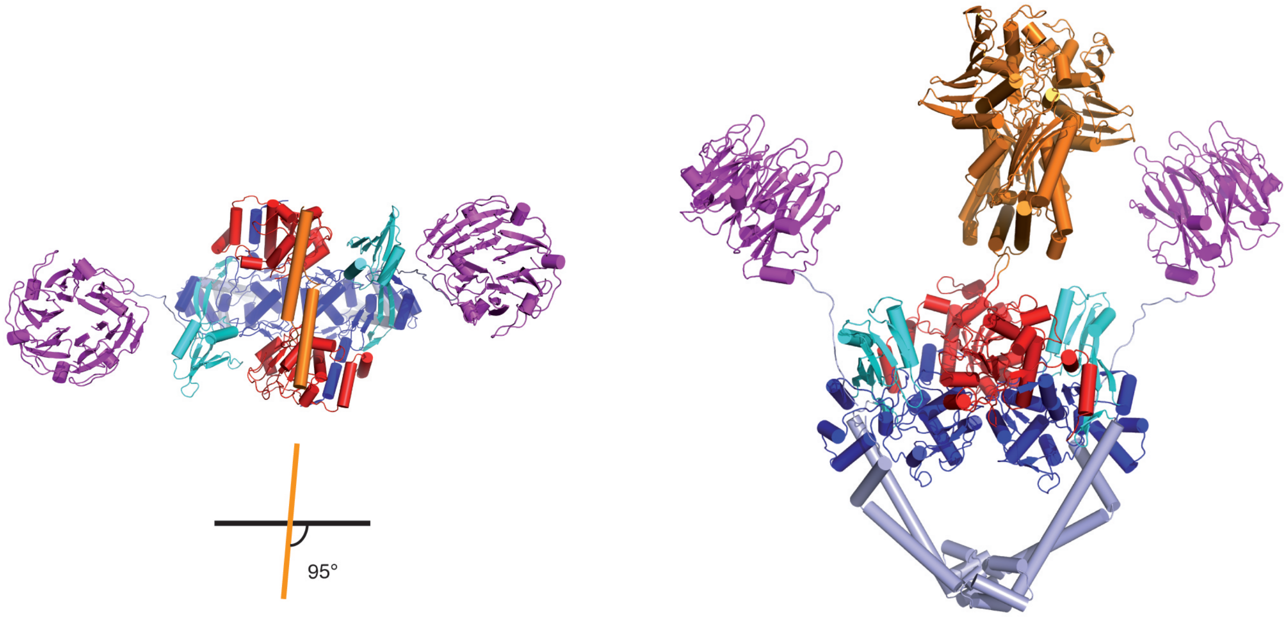

C. DNA-bound complex of *S.cerevisiae* (+nicked DNA 30bp + ADPNP)

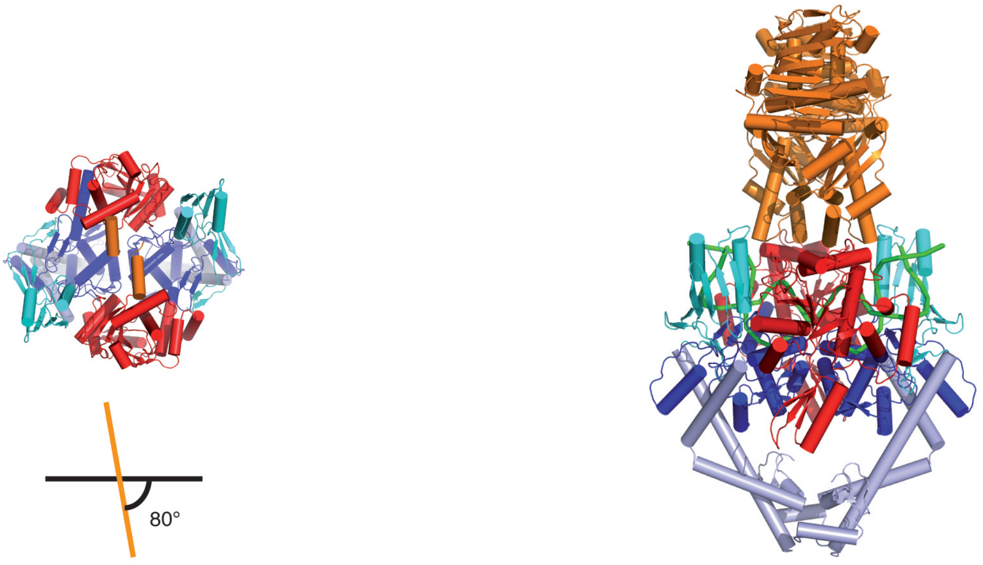

Figure S6

A.

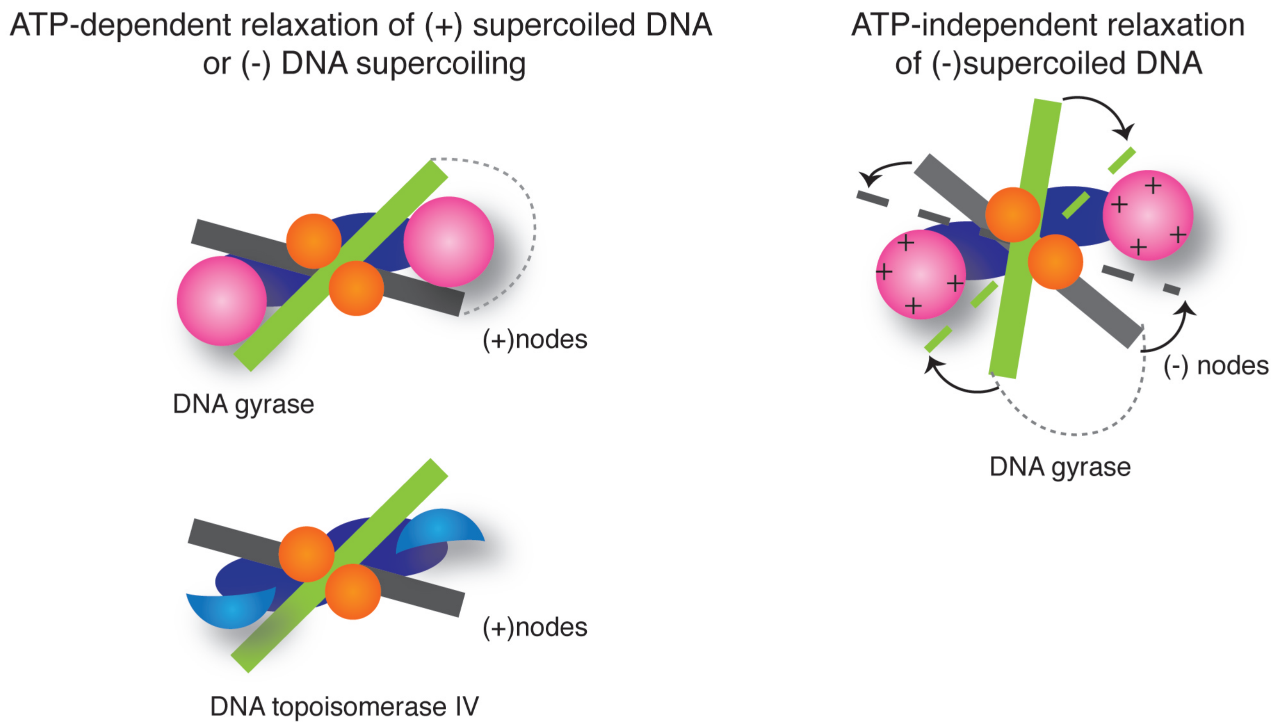
ATP-dependent relaxation of (+) supercoiled DNA
or (-) DNA supercoiling
(+)nodes
DNA gyrase
(+)nodes
DNA topoisomerase IV
ATP-independent relaxation
of (-)supercoiled DNA
(-) nodes
DNA gyrase

B.

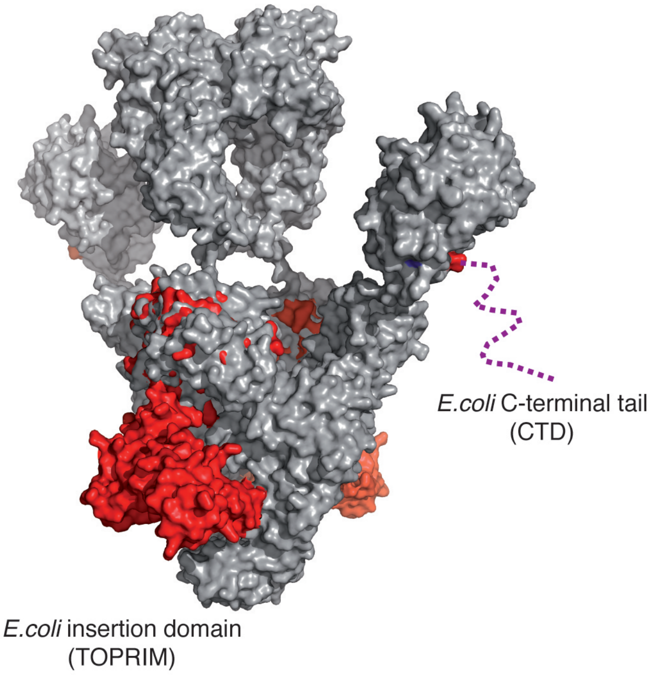
E.coli C-terminal tail
(CTD)
E.coli insertion domain
(TOPRIM)

Figure S7

A.

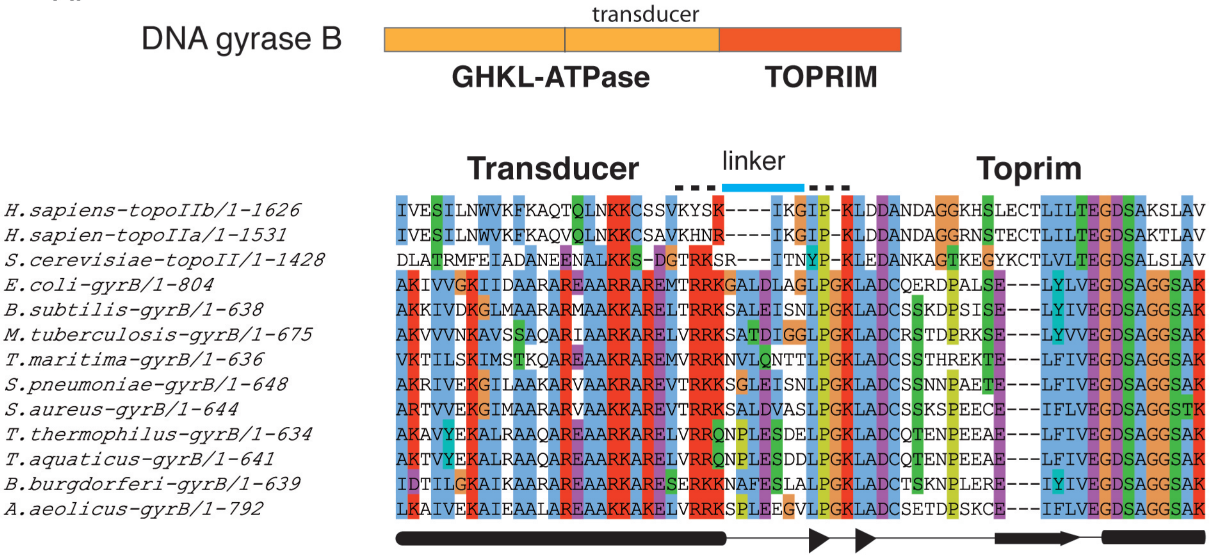

transducer
DNA gyrase B
GHKL-ATPase
TOPRIM
Transducer
linker
Toprim
H.sapiens-topoIIb/1-1626 IVESILNWVKFKAQTQLNKKCSSVKYSK----IKGIP-KLDDANDAGGKHSLECTLILTEGDSAKSLAV
H.sapien-topoIIa/1-1531 IVESILNWVKFKAQVQLNKKCSAVKHNR----IKGIP-KLDDANDAGGRNSTECTLILTEGDSAKTLAV
S.cerevisiae-topoII/1-1428 DLATRMFEIADANEENALKKS-DGTRKSR---ITNYP-KLEDANKAGTKEGYKCTLVLTEGDSALSLAV
E.coli-gyrB/1-804 AKIVVGKIIDAARAREAARRAREMTRRKGALDLAGLPGKLADCQERDPALSE---LYLVEGDSAGGSAK
B.subtilis-gyrB/1-638 AKKIVDKGLMAARARMAAKKARELTRRKSALEISNLPGKLADCSSKDPSISE---LYIVEGDSAGGSAK
M.tuberculosis-gyrB/1-675 AKVVVNKAVSSAQARIAARKARELVRRKSATDIGGLPGKLADCRSTDPRKSE---LYVVEGDSAGGSAK
T.maritima-gyrB/1-636 VKTILSKIMSTKQAREAAKRAREMVRRKNVLQNTTLPGKLADCSSTHREKTE---LFIVEGDSAGGSAK
S.pneumoniae-gyrB/1-648 AKRIVEKGILAAKARVAAKRAREVTRKKSGLEISNLPGKLADCSSNNPAETE---LFIVEGDSAGGSAK
S.aureus-gyrB/1-644 ARTVVEKGIMAARARVAAKKAREVTRRKSALDVASLPGKLADCSSKSPEECE---IFLVEGDSAGGSTK
T.thermophilus-gyrB/1-634 AKAVYEKALRAAQAREAARKARELVRRQNPLESDELPGKLADCQTENPEEAE---LFIVEGDSAGGSAK
T.aquaticus-gyrB/1-641 AKTVYEKALRAAQAREAARKARELVRRQNPLESDDLPGKLADCQTENPEEAE---LFIVEGDSAGGSAK
B.burgdorferi-gyrB/1-639 IDTILGKAIKAARAREAARKARESERKKNAFESLALPGKLADCTSKNPLERE---IYIVEGDSAGGSAK
A.aeolicus-gyrB/1-792 LKAIVEKAIEAALAREAAKKAKELVRRKSPLEEGVLPGKLADCSETDPSKCE---IFLVEGDSAGGSAK

B.

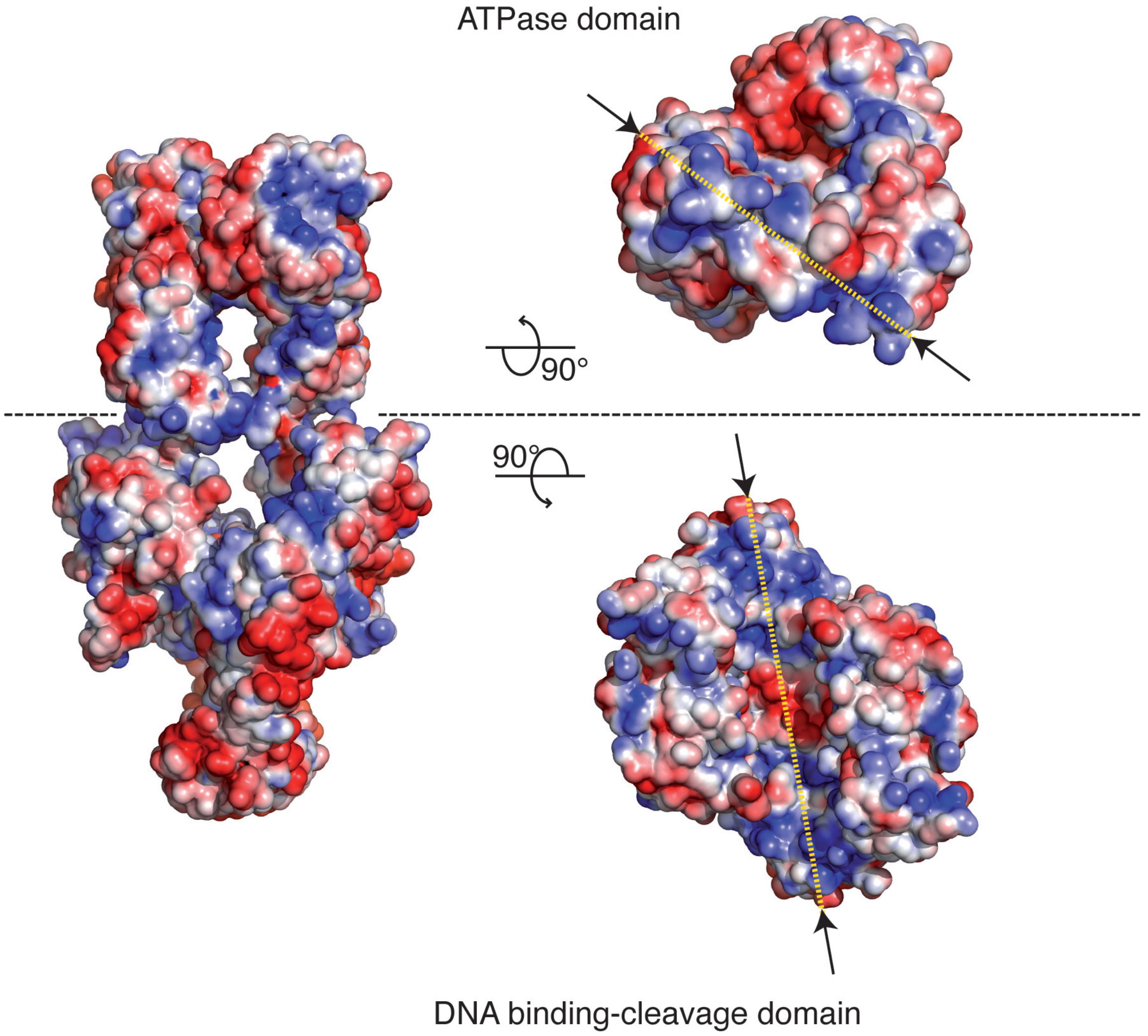

ATPase domain
90°
90°
DNA binding-cleavage domain
